# Psychobiotic *Lactobacillus plantarum* JYLP-326 relieves anxiety, depression, and insomnia symptoms in test anxious college *via* modulating the gut microbiota and its metabolism

**DOI:** 10.3389/fimmu.2023.1158137

**Published:** 2023-03-23

**Authors:** Ruizhe Zhu, Yilin Fang, Hongyu Li, Ying Liu, Jing Wei, Shuwei Zhang, Liwei Wang, Rui Fan, Lingfang Wang, Shengjie Li, Tingtao Chen

**Affiliations:** ^1^ National Engineering Research Center for Bioengineering Drugs and the Technologies, Institute of Translational Medicine, Nanchang University, Nanchang, China; ^2^ Institute of Life Science, Nanchang University, Nanchang, China

**Keywords:** *lactobacillus plantarum* JYLP-326, test anxiety, depression and insomnia, gut microbiota, untargeted metabolomics

## Abstract

**Introduction:**

Test anxiety is a common issue among college students, which can affect their physical and psychological health. However, effective interventions or therapeutic strategies are still lacking. This study aims to evaluate the potential effects of *Lactobacillus plantarum* JYLP-326 on test anxious college students.

**Methods:**

Sixty anxious students were enrolled and randomly allocated to the placebo group and the probiotic group. Both groups were instructed to take placebo and JYLP-326 products twice per day for three weeks, respectively. Thirty unanxious students with no treatments were assigned to a regular control group. The anxiety, depression, and insomnia questionnaires were used to measure students’ mental states at the baseline and the end of this study. 16S rRNA sequencing and untargeted metabolomics were performed to analyze the changes in the gut microbiota and fecal metabolism.

**Results:**

The questionnaire results suggested that JYLP-326 administration could relieve the symptoms of anxiety, depression, and insomnia in test anxious students. The gut microbiomes of the placebo group showed a significantly greater diversity index than the control group (p < 0.05). An increased abundance of *Bacteroides* and *Roseburia* at the genus level was observed in the placebo group, and the relative abundance of *Prevotella* and *Bifidobacterium* decreased. Whereas, JYLP-326 administration could partly restore the disturbed gut microbiota. Additionally, test anxiety was correlated with disordered fecal metabolomics such as a higher Ethyl sulfate and a lower Cyclohexylamine, which could be reversed after taking JYLP-326. Furthermore, the changed microbiota and fecal metabolites were significantly associated with anxiety-related symptoms.

**Conclusion:**

The results indicate that the intervention of *L. plantarum* JYLP-326 could be an effective strategy to alleviate anxiety, depression, and insomnia in test anxious college students. The potential mechanism underlying this effect could be related to the regulation of gut microbiota and fecal metabolites.

## 1 Introduction

Test anxiety, characterized by feelings of failure, tension, and worrying when an individual faces a vital test for promoting, occurs prevalently among college students. We reported that about 20%-40% of college students suffer from test anxiety in our previous study (1), and prolonged test anxiety is often accompanied by depression and insomnia, harming students’ physical and psychological health and even lead to suicide. Various examinations can represent a frequent and significant source of student’s anxiety (2). For example, the Unified National Graduate Entrance Examination in China (UNGEE) is an important opportunity for undergraduates to apply for their postgraduate studies and master’s degrees but has a reputation for cutthroat competition (3, 4). In particular, more students take this exam to escape the unstable employment market during the post-pandemic of COVID-19, aggravating the fierceness of involution of this exam and the degree of stress-related symptoms like anxiety, depression, and insomnia among examinees.

The issue of how anxious students process these exam stress-induced health threats is of great interest. Although many coping strategies have been suggested to address anxiety for students, including supplying proper nutrition (5), doing more exercises (6), and taking medicines (7), most of students tend to neglect the necessary of dietary nutrition and physical exercise due to the urgency of the UNGEE preparation. And taking medications can lead to substance dependence and induce serious adverse effects according to the FDA and scholars’ reports (8). Therefore, exploring practical and moderate interventions or therapeutical strategies is necessary for students to relieve anxiety during their preparation for examinations.

Recently, a variety of probiotics have been reported to possess effective stress-modulating and anxiolytic effects on stressed subjects *via* maintaining the intestinal homeostasis, improving mucosal and systemic immunity, and regulating the metabolism of gut microbiota, which makes those probiotics to be the Next promising living psychobiotics for alleviating psychological disorders (9–12). Additionally, the strong correlation between numerous emotional disorders (e.g., anxiety and depression) and gut microbiota dysbiosis or intestinal metabolic changes have been widely discussed, indicating an intrinsic cross-link between the gut microbiota and brain regions that has been reported as the microbiota-gut-brain axis (13, 14). Thus, targeting the gut microbiota *via* probiotics supplementation and/or faecal microbiota transplantation maybe an alternative treatment method for chronic stressed students with mental disorders like anxiety, depression, and insomnia (15). Of the reported probiotics, *Lactobacillus* are one of the most frequently researched probiotic species due to its relative abundance has been downregulated by stress and the stress-induced symptoms could be improved by the administration of single or multiple *Lactobacilli* (10, 16, 17).

*Lactobacillus plantarum*, a commonly used *Lactobacillus* exhibiting ecological and metabolic adaptability in the mammalian gastrointestinal tract niche (18), has become increasingly popular in reducing the severity of anxiety and depression in stressed animal models and crowds (19–22). Among them, *L. plantarum* JYLP-326 is an isolate that can adhere to Caco-2 cells efficiently and improve sleep disorders in an insomnia rat model (Chinese patent No.: CN 110791451 A). However, it remains unknown whether it can alleviate symptoms of anxiety, depression and insomnia in test anxious populations. Here, we enrolled 60 anxious and 30 unanxious undergraduates preparing for the approaching UNGEE to evaluate the psychological effects of JYLP-326 on exam stress-induced behaviors like anxiety, depression, and insomnia. Furthermore, changes in the gut microbiota and fecal metabolites between groups were measured by 16S rRNA high-throughput sequencing and untargeted metabolomics, respectively. These results demonstrated that the intervention of *L. plantarum* JYLP-326 is effective in alleviating exam stress-induced symptoms in college students.

## 2 Materials and methods

### 2.1 *L. plantarum* JYLP-326 and placebo products

*L. plantarum* JYLP-326 was isolated from traditionally fermented sticky rice in Bama region of Guangxi province, China, which has been preserved as patented probiotic strain in China General Microbiological Culture Collection Center (CGMCC, No. 18038) on June 27, 2019. We demonstrated that it could adhere to Caco-2 cells more efficient *in vitro* than other *L. plantarum* strains (**Supplementary Figure 1A**, **Figure S1A**), as well as JYLP-326 administration efficiently decreased the time of sleep latency and increased the total sleep time in 4-Chloro-DL-phenylalanine-induced insomnia rat model (**Figures S1B, C**). Moreover, taking JYLP-326 could restore the content of GABA in the rats’ hypothalamus to the standard level (**Figures S1D**). The probiotic and placebo (maltodextrin) products were manufactured by Zhongke Jiayi Bio Engineering Company, Shandong, China. The probiotic product contains 1 g of lyophilized JYLP-326 powder at a fixed dosage of 1.5 × 10^10^ CFU per sachet with maltodextrin as excipient. Each dose is packaged in a high barrier composite aluminum sachet and all sachets appear as light-yellow powder with identical taste. All products were manufactured under ISO9001 in China and approved as a food supplement by China Food and Drug Administration (No. SC10637078101504). Sachets were stored at 4 °C without direct sunlight exposure. The product qualities were further evaluated by the total viable count method and 16S rDNA sequencing prior to study initiation.

### 2.2 Participants and experimental design

We conducted this study to evaluate the effects of 3-week supplementation of JYLP-326 on the exam stress-induced behaviors in senior college students. Students who were nervously studying for the UNGEE during September to December 2020, a critical period for students to prepare the exam, were recruited from Nanchang University. All procedures were approved and complied with by the Institutional Ethics Committee on medical research of the Second Affiliated Hospital of Nanchang University (Examination and approval No. is Review [2020] No. (038)). All participants signed and informed consent.

**Figure 1:**
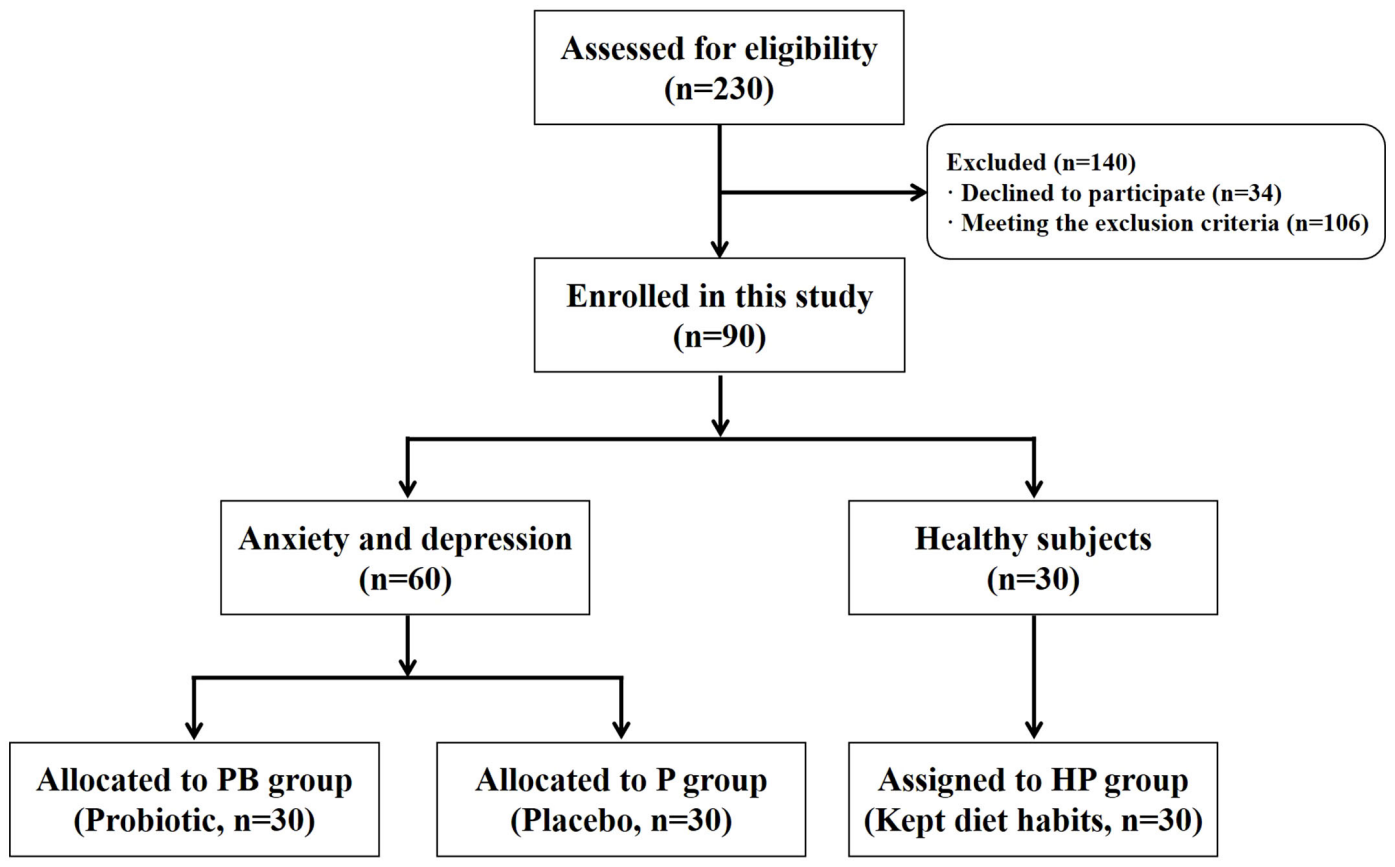
Study flow chart showing recruitment and group assignment.

Following that, a total of 230 students were enrolled to interview and completed the 14-item of Hamilton Anxiety Scale (HAMA-14), 8-item of the 8-item Athens Insomnia Scale (AIS-8) and the 17-item Hamilton Depression Rating Scale (HDRS-17); out of 230, 60 with baselines HAMA-14 for anxiety score of 8 or higher and HDRS-17 for depression score of 8 or higher were screened (1), and 30 un-anxious students with scales score of 8 or less were screened as well (**Figure 1**). The inclusion criteria were as follows: senior college students who were preparing for the coming UNGEE test; body mass index (BMI) within the healthy range; no reported severe illness (e.g., cancer, cardiac and hepatic disorders); female subjects with no hormonal contraceptives use; and willing to cooperate in completing this study. The exclusion criteria were as follows: suffering from obesity and diabetes; taking any medication regularly for certain diseases; long term smoking and drinking; taking any antibiotic or probiotics (except yogurts) within the preceding month; and other reasons that made subjects not likely to complete this study. Additionally, subjects who were experiencing failed relationships, family drama, economic pressures, and especially social anxiety disorder, were excluded as well.

Out of those 60 students with anxiety and depression facing UNGEE, 30 were selected randomly as the PB group to receive the probiotic product; another 30 received placebo product were regarded as the P group. The 30 un-anxious students were assigned as the healthy control group (HP group). The subjects in each group were of the same age and the ratio of male to female was 1:1. The subjects in PB and P groups were instructed to take probiotic and placebo product twice per day for 3 consecutive weeks with a 12-hour set time between two intakes, respectively. While students in HP group were asked to maintain their normal lifestyle and diet habits. All treatments were stopped 2 weeks before UNGEE commenced. The validated questionnaires (i.e., HAMA-14, HDRS-17, and AIS-8) were provided to determine their mental conditions at baseline (Week 0) and the end of intervention (Week 3). The subjects were instructed to collect fecal samples by themselves at home using a stool sampling tube according to the manufacture’s introduction. The first pooped fecal sample in a day were collected during 3 days after the indicated time points and stored at -80 °C immediately for further research.

### 2.3 Questionnaires

The severity of the insomnia symptoms was measured using the Chinese version of AIS-8 (23). It covers a total score ranging from 0 (denoting absence of any sleep-related problem) to 24 (representing the most severe degree of insomnia) with a cutoff score of 6 has been suggested as insomnia (24). The depressive symptoms of students were evaluated using the Chinese version of HDRS-17 (1, 25). The severity of the depressive symptoms was classified according to the following severity range of the HDRS score: normal (0–7), mild depression (8–16), moderate depression (17–23), and severe depression (> 23). The anxiety symptoms were measured using the Chinese version of HAMA-14 (1, 25), and anxiety symptom severity was classified according to the following severity range of the HAMA score: normal (0–7), mild or probable anxiety (8–14), moderate or definite anxiety (15–21), and severe anxiety (≥ 22).

### 2.4 DNA extraction and 16S rRNA sequencing

Total genomic DNA in students’ feces samples was extracted from the glass fiber filters using TIANamp Bacteria DNA Kit (QIAGEN) following the manufacturer’s instructions. The concentration and quality of the extracted genomic DNA were determined using spectrophotometer (NanoDrop, Thermo Fisher Scientific, Inc., USA). Extracted DNA was subjected to 16S sequencing at Shanghai Personal Biotechnology Cp. Ltd. The following universal 16S rRNA primers were used for the PCR reaction: 520F (5′-AYTGGGYDTAAAGNG-3′) and 802R (5′-TACNVGGGTATCTAATCC-3′) for the V4 region, and these PCR products were sequenced with an Illumina HiSeq 2000 platform. FLASH was applied to merge overlapped reads, and sequence analysis was performed using UPARSE software package. Reads with quality scores lower than 20, ambiguous bases, and improper primers were discarded prior to clustering. Simultaneously, chimaeras were checked and eliminated during clustering. The resulting high-quality sequences were clustered into operational taxonomic units (OTUs) at 97% similarity. According to the annotation of taxa, the relative abundance of total bacteria in each sample was categorized into different classification levels (Phylum, Class, Order, Family, and Genera). The alpha diversity index (Shannon index and Chao1 index) was calculated, and the beta diversity index (principal component analysis, PCoA) was mapped in QIIME2 software. The raw reads were deposited in the Sequence Read Archive (SRA) database of NCBI (PRJNA883039).

### 2.5 Fecal metabolites analysis

Metabolomics in feces were analyzed at Shanghai Personal Biotechnology Cp. Ltd using an UHPLC (1290 Infinity LC, Agilent Technologies) coupled to a quadrupole time-of-flight (AB Sciex TripleTOF 6600). Stool weighing 100 mg was added with 400 ml extract of methanol-acetonitrile-water (2:2:1, vol/vol/vol), vortexed and centrifuged at 14,000 g for 20min, and the supernatant was collected for further measurement. For HILIC separation, samples were analyzed using a 2.1 mm × 100 mm ACQUIY UPLC BEH 1.7 µm column (waters, Ireland). In both ESI positive and negative modes, the mobile phase contained A=25 mM ammonium acetate and 25 mM ammonium hydroxide in water and B= acetonitrile. For RPLC separation, a 2.1 mm × 100 mm ACQUIY UPLC HSS T3 1.8 µm column (waters, Ireland) was used. In ESI positive mode, the mobile phase contained A= water with 0.1% formic acid and B= acetonitrile with 0.1% formic acid; and in ESI negative mode, the mobile phase contained A=0.5 mM ammonium fluoride in water and B= acetonitrile. The raw MS data (wiff.scan files) were converted to MzXML files using ProteoWizard MSConvert before imported into freely available XCMS software. For peak picking, the following parameters were used: centWave m/z = 10 ppm, peak width = c (10, 60), prefilter = c (10, 100). For peak grouping, bw = 5, mzwid = 0.025, minfrac = 0.5 were used. CAMERA (Collection of Algorithms of MEtabolite pRofile Annotation) was used for annotation of isotopes and adducts.

After normalized to total peak intensity, the processed data were analyzed by R package, where it was subjected to multivariate data analysis, including Pareto-scaled principal component analysis (PCA) and orthogonal partial least-squares discriminant analysis (OPLS-DA). The 7-fold cross-validation and response permutation testing were used to evaluate the robustness of the model. The variable importance in the projection (VIP) value of each variable in the OPLS-DA model was calculated to indicate its contribution to the classification. Metabolites with the VIP value > 1 were further applied to Student’s t-test at the univariate level to measure the significance of each metabolite. The *p* values less than 0.05 were considered as statistically significant. The raw data on fecal metabolomics were deposited in the public databases of Metabolomics Workbench (ST002475) and Metabolights (MTBLS7119).

### 2.6 Statistical analysis

Spearman correlation analysis was performed by R package and used to determine the correlation between anxiety/depression scores and the main changed microbes, as well as the correlation between microbes and the differential metabolites (26). Statistical analysis was performed using Prism software version 8.0 (GraphPad Software, San Diego, CA, USA). Data was shown as the mean ± SD. Statistical significance was analyzed using a One-way analysis of variance (ANOVA) followed by Tukey’s multiple comparison test. Error probabilities of *p <*0.05 were considered statistically significant.

## 3 Results

### 3.1 JYLP-326 alleviates anxiety, depression, and insomnia symptoms in students

There were no significant differences in the background features of age and BMI among the enrolled students in these groups (*p* > 0.05, **Table 1**). The HAMA-14, HDRS-17, and AIS-8 questionnaires were used to assess the subjects’ mental states at the baseline (Week 0) and the end of the treatment (Week 3). Compared to the healthy students, subjects in the PB and P groups exhibited obvious anxiety and depression at the baseline (*p* < 0.001, **Table 1**). In the HP group, both HAMA-14 anxiety and HDRS-17 depression scale scores dramatically increased from 4.07 ± 1.51 and 2.83 ± 1.39 at Week 0 to 7.63 ± 2.40 and 6.83 ± 2.97 at Week 3, respectively, indicating that healthy students’ mood gradually turned to mild anxiety and depression as the exam approached (**Table 1**). These two indicators in the P group were marginally enhanced at the end of this treatment, whereas they were significantly decreased in the PB group. Moreover, the severity of insomnia in the subjects was positively correlated with the level of anxiety and depression. Collectively, these results suggested that probiotic JYLP-326 supplement can alleviate the physiological disorders and states of subjects facing chronic stress, including anxiety and depression as well as insomnia.

**Table 1:** Characteristics of subjects enrolled in this study and efficacy outcomes of tests as assessed *via* these scales.

	HP group (n=30)		*p* value*	P group (n=30)		*p* value*	PB group (n=30)		*p* value*
	Week 0	Week 3		Week 0	Week 3		Week 0	Week 3	
Age	22.25 ± 0.11		/	22.50 ± 0.25		/	22.30 ± 0.25		/
BMI	21.20 ± 2.48		/	20.83 ± 2.31		/	21.07 ± 2.67		/
Female: male (n: n)	1: 1 (15.00: 15.00)		/	1: 1 (15.00: 15.00)		/	1: 1 (15.00: 15.00)		/
HAMA-14^a^	4.07 ± 1.51^A^	7.63 ± 2.40****^A^	< 0.0001	13.27 ± 3.64^B^	15.30 ± 3.71*^B^	0.0362	13.93 ± 5.01^B^	10.57 ± 3.43**^C^	0.0036
HDRS-17^b^	2.83 ± 1.39^A^	6.83 ± 2.97****^A^	< 0.0001	12.23 ± 4.59^B^	14.07 ± 2.07^nsB^	0.1083	12.37 ± 5.41^B^	8.17 ± 4.13***^A^	0.0003
AIS-8^c^	2.33 ± 1.06^A^	5.07 ± 1.93***^A^	0.0002	8.27 ± 3.19^B^	9.53 ± 3.45^nsB^	0.1455	8.07 ± 2.85^B^	6.47 ± 2.15*^A^	0.0171

Data was shown as mean ± SD; n refers to number; BMI, baseline body mass index.

a The 14-item Hamilton Anxiety Scale (HAMA-14) was used to assess the anxiety symptoms.

b The 17-item Hamilton Depression Rating Scale (HDRS-17) was used to evaluate the depressive symptoms.

c The 8-item Athens Insomnia Scale (AIS-8) was used to assess the insomnia symptoms.

*Intragroup, compared to baseline (Week 0), *p < 0.05, **p < 0.001, ***p < 0.001, ****p < 0.0001. ns refers to no significance.

Capital letters refer to Tukey’s multiple comparisons test at intergroup, p < 0.001.

### 3.2 JYLP-326 has impacts on the gut microbial diversity

**Figure 2:**
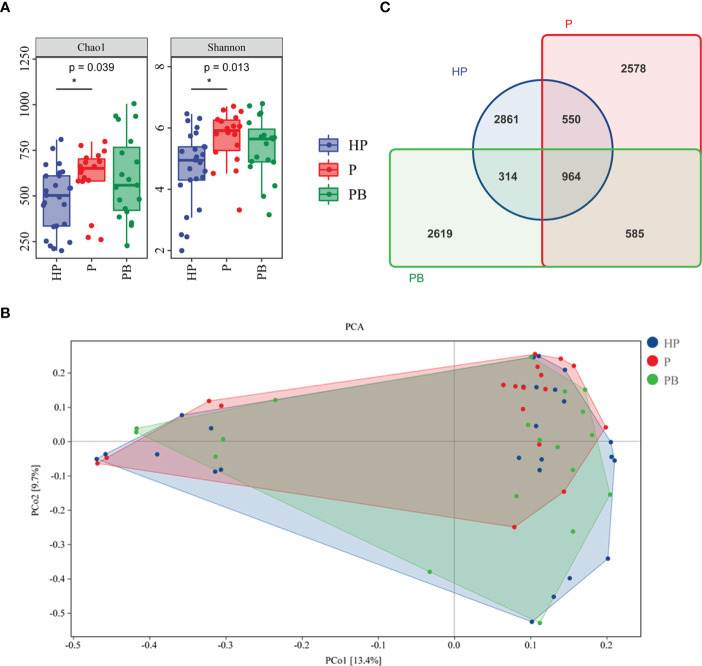
Effects of *L. plantarum* JYLP-326 on the gut microbial diversity. **(A)** Chao1 and Shannon indices of the gut microbiota. **(B)** PCoA analysis of gut microbiota. **(C)** Venn diagram of gut microbial species. HP, the normal control group without any treatmeat; P, the P group taking placebo; PB, the PB group taking probiotic *L. plantarum* JYLP-326. ∗ *p* < 0.05.

All subjects were invited to provide fecal samples voluntarily. But we finally collected 25 fecal samples from the HP group, 20 from P group and 20 from PB group to perform 16S rRNA high-throughput sequencing analysis. And 2 samples in P group and 1 sample in PB group failed to establish sequencing library. There was a difference in α-diversity indices of gut microbiota among these three groups as the Chao 1 and Shannon index presented, suggesting that test anxiety might disturb the balance of human gut microbiota (**Figure 2A**). In particular, the Chao 1 and Shannon index in the P group were marginally greater than that of in HP group, while JYLP-326 could restore the diversity of gut microbiota that was disturbed by test anxiety (**Figure 2A**). In addition, the PCoA analysis demonstrated that the β-diversity of human gut microbiota in the PB group was similar with that in the HP group but slightly different with that in P group, which further suggested that test anxiety had impact on the structure of human gut microbiota and probiotic JYLP-326 could remodel the gut microbial profile (**Figure 2B**). Moreover, a total of 10, 471 OTUs were identified in all samples, but less than 10% (964/10, 471) of OTUs were common to the three groups, and only 32.37% of OTUs in the P group and 28.51% of OTUs in the PB group were shared by the HP group, indicating that test anxiety may affect species types inhabited in the gut and that treatment with JYLP-326 alone can hardly reshape this change (**Figure 2C**).

### 3.3 Effects of JYLP-326 on the fecal microbial composition

**Figure 3:**
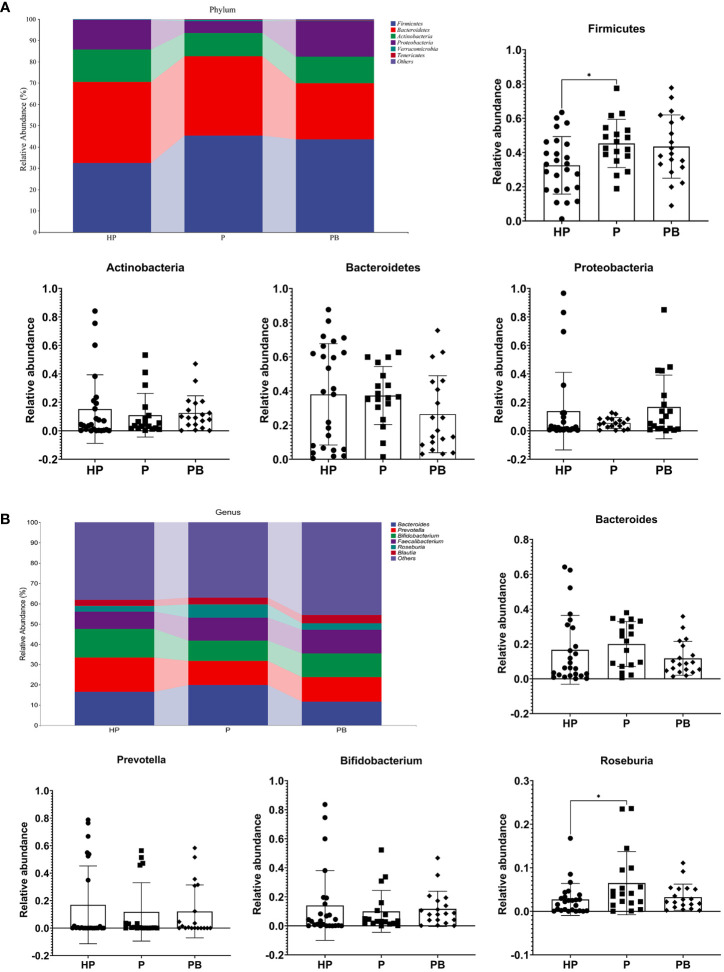
Effects of JYLP-326 on the gut microbial composition. **(A)** The relative abundance of gut bacteria at the phyla level. **(B)** The relative abundance of gut bacteria at genus level. HP, the normal control group without any treatment; P, the P group taking placebo; PB, the PB group taking JYLP-326. ∗ *p* < 0.05.

At the phylum level, Firmicutes, Bacteroidetes, Actinobacteria and Proteobacteria were the most common populations in these groups, and they accounting for 99.76%,99.53%, and 99.30% of the total sequencing results in these three groups, respectively (**Figure 3A**). Remarkably, the relative abundance of Firmicutes was 45.28% in the P group, which was significantly higher than that in the HP group (32.51%, p = 0.041), whereas it could be restored slightly by the administration of JYLP-326. In contrast, the relative abundance of Actinobacteria and Proteobacteria in the P group (10.96% and 5.45%) were lower than that in the HP (15.26% and 13.83%) and PB group (12.44% and 16.76%). Additionally, the content of Bacteroidetes was not influenced by test anxiety as its relative abundance was very similar between P and HP group (38.04% *vs* 37.13%), yet it could be reduced by treatment with JYLP-326.

At the genus level (**Figure 3B**), the fecal microbial spectrum of students in the P group was less rich in *Prevotella* (11.80%) and *Bifidobacterium* (9.98%) than that in the HP group (16.89% and 14.05%), but was relative rich in *Bacteroides* (19.90%) and *Roseburia* (6.47%) than that in the HP group (16.61% and 2.73%). And their relative abundance in the PB group was 12.12%, 11.69%, 11.73%, and 3.25%, respectively. These results illustrated that treatment with JYLP-326 could partly reverse the trend of change in gut microbiome to the standard state. Of note, the abundance of *Roseburia* in the P group was dramatically higher (*p* = 0.0397) than that of the HP group, whereas taking probiotic JYLP-326 could eliminate the change trend of this genera. Collectively, these results indicated that the administration of JYLP-326 could remodel the test anxiety-induced changes in the composition of gut microbiota, particularly in decreasing *Bacteroides* and *Roseburia* and increasing *Prevotella* and *Bifidobacterium*.

### 3.4 Identification of changed fecal metabolites among each group

**Figure 4:**
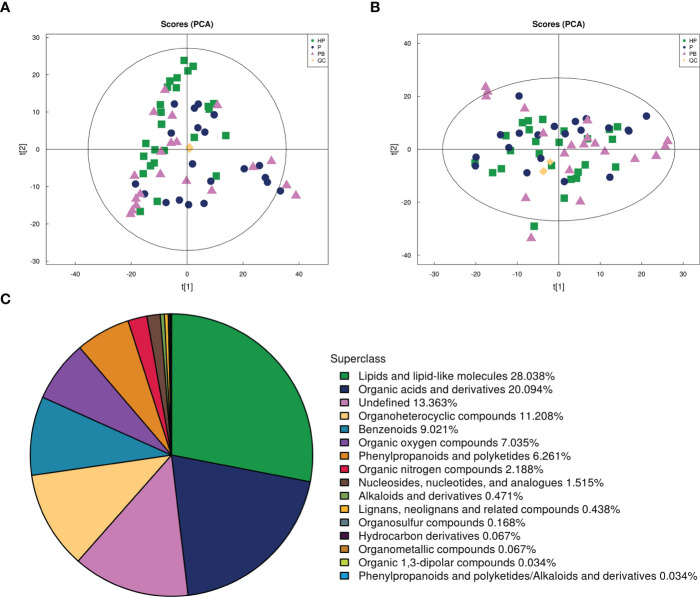
Fecal metabolome profiling among groups. **(A)** PCA plots in ESI+ model. **(B)** PCA plots in ESI- model. **(C)** Superclass of the identified fecal metabolites. HP, samples of the normal control group (n=25, green box); P, samples of the P group (n=20, purple circle); PB, samples of the PB group (n=20, pink triangle); QC, the quality control samples (n=7, yellow diamond).

The quality control (QC) samples were inserted into the sample cohorts to monitor and evaluate the stability of the test system and the reliability of experimental data. The response intensity and retention time of each chromatographic peak of all QC samples were almost overlapped in their total ion chromatograms (**Figure S2**), and all QC samples clustered together under the ESI- and ESI+ modes (**Figures 4A, B**), suggesting that the obtained data for analysis was reliable and the analytical system was stable. Besides, there was no obvious separation among these three groups based on the PCA analysis, indicating that test anxiety and probiotic JYLP-326 seemed to have no significant impacts on the changes of fecal metabolic profiles. A local self-built standard product database library was adopted to search and match the detail information of metabolites in the samples, and a total of 2, 791 metabolites (1, 818 under the ESI+ modes, and 1,153 under the ESI- modes) were identified at the level 2 of the new confidence level of compound annotations. According to their chemical taxonomy information, 28.04% of metabolites were classified into the superclass of lipids and lipid-like molecules, 20.09% were organic acids and derivatives, and 13.36% were belonged to the undefined one (**Figure 4C**).

### 3.5 JYLP-326 promotes the fecal metabolic rearrangement in anxious students

**Figure 5:**
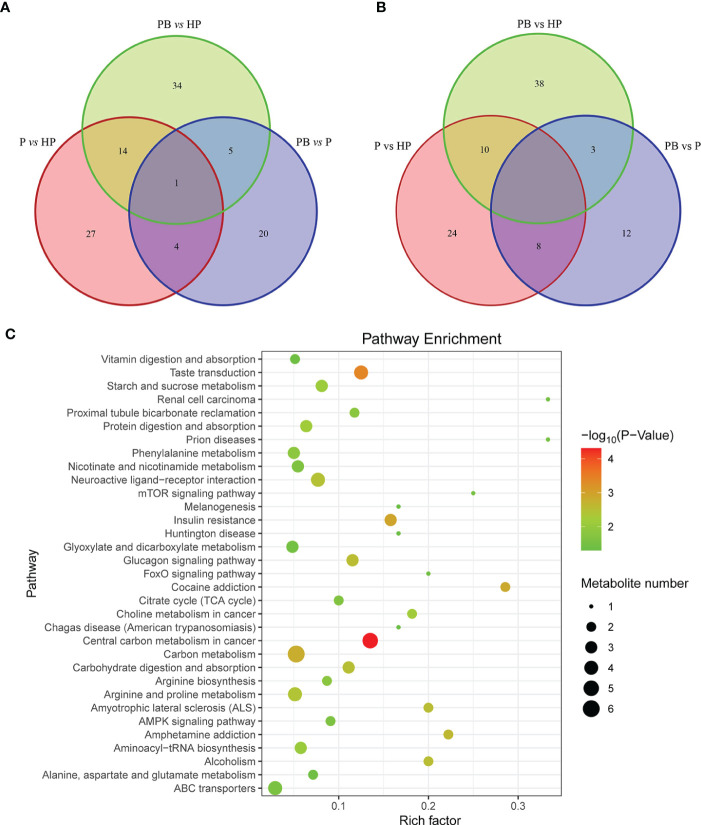
Pathway enrichment analysis of differential metabolites. **(A, B)** Venn diagram of differential metabolites in ESI+ and ESI- modes. A total of two hundred differential metabolites were shown in the Venn diagrams. **(C)** KEGG enrichment analysis of these changed metabolites. The scatter diagram shows the enrichment of differential metabolites in various pathways.

To explore the differential metabolites among these groups, we used OPLS-DA models to compare the differential variables between groups, and the value of variable importance for the projection (VIP) obtained by OPLS-DA (VIP > 1) and p value (*p* < 0.05) as the criteria to screen the changed metabolites between two groups. A distinct separation between groups under the ESI- modes (**Figures S3A–C**) and ESI+ modes (**Figures S3D–F**) was observed, suggesting that OPLS-DA models could distinguish between samples effectively. Furthermore, we selected the variables with the value of fold change (FC > 1or < 1) and P value < 0.05 to screen the potential metabolites. The Venn diagram showed that a total of 95 variables of metabolites under the ESI- mode and 105 under the ESI+ mode were screened (**Figures 5A, B**). The detailed information of these metabolites was listed in **Supplementary Tables 1**, **2**. The KEGG enrichment analytical scatter diagram showed that these variables were significantly enriched in the major pathways of Carbon or energy metabolism, Insulin resistance, neuroactive ligand-receptor interaction, and Vitamin digestion and absorption (**Figure 5C**).

Meanwhile, there were 13 variables in the intersection between P *vs*. HP and PB *vs*. P (**Table 2**), which formed a subset of variables related to probiotic JYLP-326. Compared to the HP group, the relative levels of 8 variables (i.e., L-gulono-1,4-lactone, Ethyl sulfate, Quinate, D-glucarate, 1,2-propanediol, 3-(1,3-benzodioxol-5-yl)-, 3-Methoxy-4-Hydroxyphenylglycol Sulfate, Betaine, and Trigonelline) increased significantly in the P group (FC > 1 and *p* < 0.05), and the relative levels of 5 variables (i.e., Fenpropidin, Palmitoyl 3-carbacyclic phosphatidic acid, 5 beta-androstan-3 alpha-ol-17-one Sulfate, Biocytin, and Cyclohexylamine) decreased obviously (FC < 1 and *p* < 0.05). Whereas, these variables could be reversed to the standard level after JYLP-326 treatment, with the exception of the metabolite of Fenpropidin. These results led us to conclude that these variables could be the metabolic markers for probiotic JYLP-326 against test anxiety in students.

**Table 2:** The detailed information of 13 significantly changed metabolites among HP, P, and PB groups ^*^.

Model	Specific Name	m/z ^a^	rt(s) ^b^	P *vs* HP			PB *vs* P		
				VIP ^c^	FC ^d^	*p*-value	VIP ^c^	FC ^d^	*p*-value
ESI-	L-gulono-1,4-lactone	195.05081	387.357	4.27301971	7.979424421	0.017582821	3.906341727	0.113596458	0.029565625
ESI-	Ethyl sulfate	124.99066	38.071	1.882300315	7.075238951	0.013742364	1.292556755	0.136934342	0.025845551
ESI-	Quinate	191.05593	345.186	3.173086907	5.276268253	0.002457568	1.455844085	0.362811164	0.039252262
ESI-	D-glucarate	191.01949	263.965	2.369376541	3.646624883	0.008140489	2.386902382	0.172475162	0.027773458
ESI-	1,2-propanediol, 3-(1,3-benzodioxol-5-yl)-	195.05076	424.711	1.835589385	3.597884209	0.030419397	1.721596256	0.205156794	0.024078796
ESI-	3-Methoxy-4-Hydroxyphenylglycol Sulfate	263.02291	50.099	2.906884199	3.453605621	0.003080449	3.174853095	0.259107401	0.003582272
ESI+	Betaine	118.086	264.934	17.57192332	2.739920259	0.001233787	15.37404912	0.503744169	0.014949984
ESI+	Trigonelline	138.05419	283.845	2.373539161	2.186249216	0.019879179	3.007366424	0.378027272	0.00426738
ESI+	Fenpropidin	274.27371	60.342	4.551799883	0.692642154	0.007369509	2.088344672	0.703853494	0.019033832
ESI+	Palmitoyl 3-carbacyclic phosphatidic acid	413.26432	116.372	3.758160942	0.418147894	0.017807482	3.366001471	1.936188022	0.038522023
ESI-	5 beta-androstan-3alpha-ol-17-one Sulfate	369.17338	33.0335	5.632922694	0.399757525	0.038920948	6.557939453	2.900713193	0.004282649
ESI-	Biocytin	371.18864	35.274	2.481678326	0.343132465	0.027963888	3.599344931	3.172244195	0.001949106
ESI+	Cyclohexylamine	100.11085	71.223	4.009630411	0.289296434	0.040504798	5.697031322	3.784354688	0.03088384

^*^ The relative abundance of these metabolites in each group has been deposited in the public databases of Metabolomics Workbench (ST002475) and Metabolights (MTBLS7119).

a m/z means the mass-to-charge ratio of each metabolite.

b rt(s) refers to the retention time of metabolite on the chromatography, i.e., the peak time, which is expressed in second.

c VIP, the variable Importance for projection; the larger of the value, the more important.

d FC means fold changes of metabolite in the compared groups.

### 3.6 Correlation analysis among metabolome, microbiome, and phenotypes

**Figure 6:**
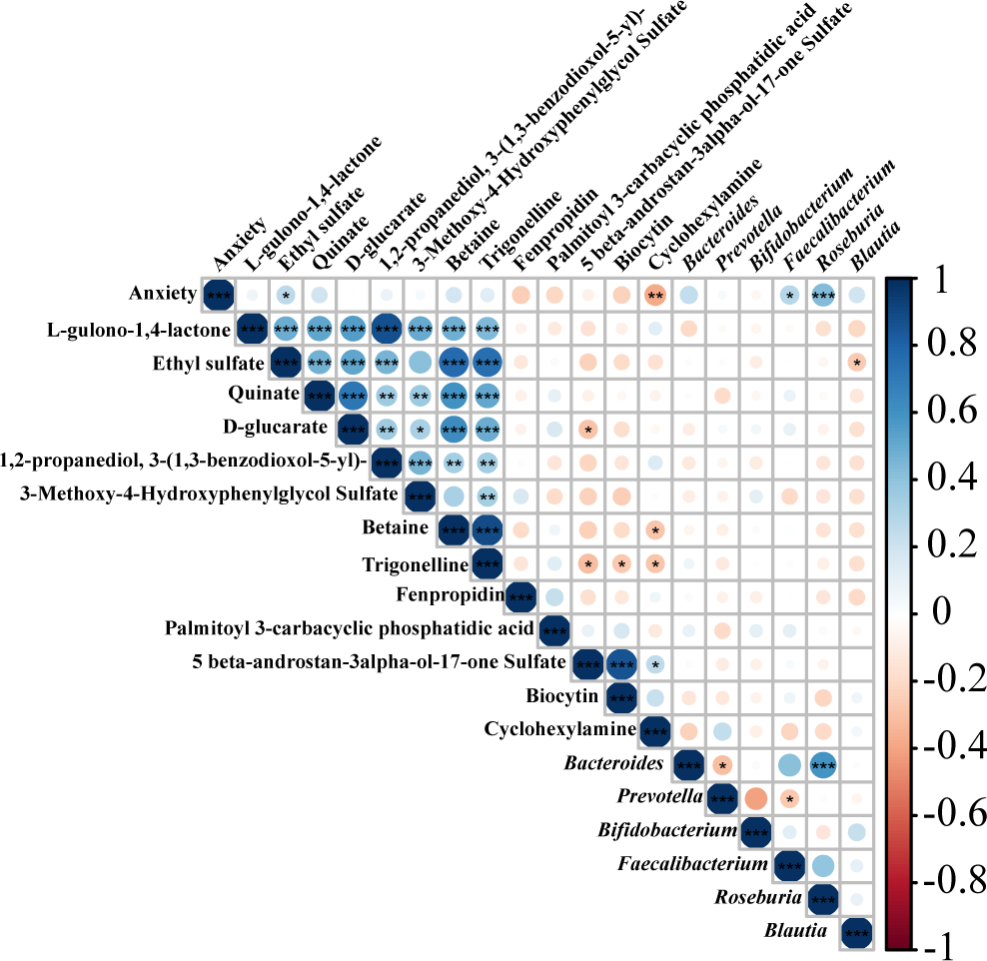
Spearman correlation analysis among anxiety-behavior, gut microbiota, and fecal metabolites. Spearman’s rank correlation coefficient among anxiety symptom scores, 13 metabolites in **Table 2** and top 6 relative abundance of gut microbiota. P values are depicted in red and blue, where red refers to a negative correlation and blue refers to a positive correlation. ∗*p* < 0.05, ∗∗*p* < 0.01, ∗∗∗*p* < 0.001.

Then, Spearman correlations were carried out to investigate association among the gut microbiota genera (top 6), 13 differential metabolites, and anxiety symptom (**Figure 6**). We observed that *Bacteroides*, *Faecalibacterium*, *Roseburia*, and *Blautia* were positively correlated with the exam stress-induced test anxiety in students. Notably, the genera *Faecalibacterium* and *Roseburia* were significantly associated with each other and positively correlated with *Bacteroides* that was negatively correlated with *Prevotella*. And the genera *Prevotella* was negatively correlated with *Bifidobacterium* and *Faecalibacterium* as well. Among the 13 metabolites, eight were positively correlated with each other but negatively correlated with the remaining five ones. Remarkably, there was a remarkable negative correlation between Cyclohexylamine and anxiety symptom, and a significant positive correlation between Ethyl sulfate and exam stress-induced anxiety. Moreover, the 13 metabolites seemed to be negatively correlated with *Roseburia* and *Blautia*. In addition, the correlations among depression/insomnia, the 13 metabolites, and 6 changed gut microbes were similar with that of anxiety (**Figures S4A, B**). These results suggested that the changes of gut microbiota and its metabolism were associated with exam stress-induces test anxiety, depression, and insomnia in students.

## 4 Discussion

The academic examination is the primary source of brief naturalistic stress for college students, which has been used frequently as a stress model to investigate the psychological stress responses together with gut microbiota changes and to evaluate the influences of probiotics or prebiotics on stress-induced complications (27). In this study, healthy college students undergoing UNGEE characterized by its fierce involution were enrolled to assess the potential effects of probiotic *L. plantarum* JYLP-326 on stress-related symptoms. Results showed that the daily supplementation of probiotic JYLP-326 (twice per day) for three weeks significantly relieved college students’ anxiety/depression and insomnia. Mechanistically, probiotic treatment could drive shifts in the diversity and composition of gut microbiota disturbed by the stress event, which was accompanied by their functional metabolic changes. The current data further indicated a bidirectional communication pathway between gut microbiota and brain [i.e., gut-brain axis (28)], as well as validated the beneficial psychological effects of probiotics in anxious subjects (29, 30).

The anxiety, depression, and insomnia levels were measured by the most commonly used mental health questionnaires, such as HAMA-14, HDRS-17, and AIS-8 (1, 23). The students in the P and PB groups showed moderate anxiety and depression accompanied by mild sleep disorder at the baseline. The scale scores were remarkably gained at the end of this study in the HP and P groups, suggesting that the levels of these indicators were intensifying and were set to get even worse during the approaching UNGEE examination. Notably, the probiotic JYLP-326 treatment significantly reduced depression and sleep quality to a borderline between regular and moderate, and protected against the progress of anxiety to be worse (**Table 1**). These data corroborated well with previous results that *L. plantarum* single strain (e.g., P8, PS128, DR7) reduced stress, anxiety, and insomnia in stressed subjects (20, 21, 23, 31). Other *Lactobacillus* species like *L. casei* (Shirota) (27), *L. rhamnosus* (16), and multi-strain products contained *L. plantarum* [e.g., UBLP40 (17), R1012 (11), SK321 (32)] also have been reported to modulate psychiatric disorders and stress-induced behaviours. These findings indicated probiotics like *Lactobacillus* had potential impacts on reducing test anxiety and its related symptoms.

Considering evidence suggested that probiotic interventions could balance microbial populations thereby create healthy intestinal homeostasis to support the life system. There is an assumption that a higher gut microbiota diversity generally means a better gut environment, which has been proved by several conditions (e.g., cancers, gastrointestinal disorders, and stress-related conditions) accompanied by a reduced gut microbial diversity (22). However, the data shown here were incongruent with those observed findings, because we found a significantly increased α-diversity of the gut microbiota (i.e., the Shannon and Chao1 index) and a minor alteration of β-diversity (PCA analysis) in the placebo group compared to the healthy control group (**Figure 2**). Another scholar also thought that measurement of diversity is not necessarily related to the answer of community outcomes, but a starting point for further inquiry of ecological mechanisms (33).

Our results showed that the content of *Bacteroides*, *Faecalibacterium*, and *Roseburia* genera increased obviously along with the exacerbation of stress-related diseases, while *Prevotella* and *Bifidobacterium* genera decreased; whereas JYLP-326 treatment partly reversed the drifts of these bacteria (**Figure 3**). Some *Bifidobacterium* and *Faecalibactreium* specific species like *B. adolescentis* (34), *B. longum* (35), and *F. prausnizii* (36) have been found to be upregulated and *R. faecis* (22) downregulated after taking *L. plantarum* in stressed subjects. *Lactobacillus*, an important genus in the gut microbiota, has also been reported to correlate significantly with mental health and possess anxiolytic and antidepressant abilities (37). However, the relative abundance of *Lactobacillus* was so extremely low that we hardly found its alteration among groups. The possible explanation for this observation was that the redundancy procedures of 16S sequencing lost the information of Lactobacillus, and even the isolated *L. plantarum* JYLP-326 could not colonize in the gut. Remarkably, correlation analysis data presented here further found that *Roseburia* was positively correlated with stress-related symptoms (**Figure 6**), similar to the results of another study (22). These results supported that specific gut microbial species and their relative richness play vital roles in protecting against the progress of stress-induced disorders.

Furthermore, gut microbial metabolic actions or gut microorganism-originated metabolites are the major pathways promoting the communication between gut and brain *via* the microbiota-gut-brain axis (37). Short-chain fatty acids (SCFAs) (38), bile acids (39), and some neurotransmitters such as γ-amino butyric acid (GABA) (40) and dopamine (41) were commonly metabolites potentially involved in this communication. Here, two hundred differential fecal metabolites were identified by untargeted metabolism analysis among these groups, which were enriched in pathways like Vitamin digestion and absorption, Neuroactive ligand-receptor interaction, Insulin resistance, and Carbon or energy metabolism (**Figure 5**). The gut microbiota synthesized Vitamin (namely Vitamin K2) could be a neuroprotective agent to restore Parkinson’s disease (PD)-associated mitochondrial defects in drosophila (42). Other researchers and we suggested that insulin resistance is closely linked to neurodegenerative diseases like PD, and impaired brain insulin signaling is a feature contributing to neuronal dysfunction in these disorders (43, 44). These results further suggested that stress-induced disorders were associated with disturbances of fecal metabolomics.

Among them, 13 variables formed a subset related to probiotic JYLP-326 treatment (**Table 2**), which almost seemed to be negatively correlated with the disturbed gut microbiome except for the Cyclohexylamine that was positively related to *Prevotella* (**Figure 6**). In addition, Cyclohexylamine was found to be negatively associated to anxiety/depression symptoms. In contrast, Ethyl sulfate was positively related to these disorders. The nitrogen-containing heterocyclic moieties and their derivatives like Cyclohexylamine have proven success in treating depression (45). And Ethyl sulfate has been considered a biomarker in the serum of alcohol addiction that will cause a series of psychotic disorders (46). Although the alterations of the frequently mentioned SCFAs and GABA in the fecal metabolites were not observed in this study, our results provided some novel compounds implicated in the development of stress-related symptoms, which would be modulated by the administration of probiotics like *L. plantarum* JYLP-326. Further studies should be conducted to illustrate the precise functions of those variables involved in the efficacy of probiotics on stress-related symptoms like anxiety and depression.

Although the potential role of JYLP-326 in remodeling the disturbed gut microbiota has been illustrated in this study, it’s still unclear whether this efficacy was caused by probiotic administration or individual differences among subjects. It would be better to take into account the impact of gut microbiome heterogeneity on the results of gut microbiota and its metabolism by collecting fecal samples before the treatments as additional control groups for further comparative analysis. Moreover, the underlying mechanisms of JYLP-326 in relieving the test anxiety-related mental disorders also should be explored deeply by using anxious/depression animal models and advanced molecular techniques, particularly in how to link the microbiota-gut-brain axis with the biological functions of probiotic JYLP-326 in the future, a critical issue on the applying of probiotics in the clinic.

## 5 Conclusions

In Conclusion, the present study observed that the probiotic JYLP-326 administration could significantly alleviate the anxiety/depression and insomnia symptoms in test anxious college students. The structure and composition of the gut microbiota were altered by the pressure to pass a vital examination with fierce involution, as well as the same to the changes in fecal metabolites. Specifically, JYLP-326 treatment protected against this exam stress-induced dysbiosis of the gut microbiota and the disturbances of fecal metabolomic. Furthermore, the changed gut microbiota genera and fecal metabolites were closely associated with stress-related symptoms like anxiety/depression and insomnia, indicating they might be regarded as biomarkers for diagnosing and treating stress and anxiety disorders.

## Data availability statement

The datasets presented in this study can be found in online repositories. The names of the repository/repositories and accession number(s) can be found below: PRJNA883039 (SRA) and ST002475 (metabolomicsworkbench.org).

## Ethics statement

The study was approved by the Institutional Review Board (or Ethics Committee) on medical research of the Second Affiliated Hospital of Nanchang University (Examination and approval No. is Review [2020] No. (038)). The patients/participants provided their written informed consent to participate in this study.

## Author contributions

Conceptualization, TC. Methodology, RZ and SL. Software, RZ. Validation, TC and SL. Formal analysis, SL. Investigation, YF, HL, SZ, RF and YL. Resources, JW. Data curation, RZ. Writing—original draft preparation, SL and RZ. Writing—review and editing, SL, TC, SZ and LWW. Visualization, RZ and SL. Supervision, TC and JW. Project administration, TC. Funding acquisition, TC, SL and LFW. All authors contributed to the article and approved the submitted version.
