# Supplementary material for: Psychobiotic Lactobacillus plantarum JYLP-326 relieves anxiety, depression, and insomnia symptoms in test anxious college via modulating the gut microbiota and its metabolism
Source: Front Immunol. 2023 Mar 23;14:1158137. doi: 10.3389/fimmu.2023.1158137 (PMC10077425; doi:10.3389/fimmu.2023.1158137)
Supplement: Supplementary file 1 [file Table_1.docx]

| Table S1. Fecal metabolites with significant differences under ESI- mode between groups | | | | | | |
| --- | --- | --- | --- | --- | --- | --- |
| P vs. HP | | | | | | |
| **adduct** | **Name** | **VIP** | **Fold change** | **p-value** | **m/z** | **rt(s)** |
| [M-H]- | Indoxyl sulfate | 4.305678589 | 17.82167307 | 0.033159659 | 212.00275 | 32.698 |
| [M-H]- | Aleuritic acid | 3.244572887 | 14.61958233 | 0.04596689 | 303.19961 | 33.927 |
| [M-H]- | 5'-phosphoribosyl-5-amino-4-imidazolecarboxamide (aicar) | 1.118585066 | 10.29899398 | 0.024864274 | 337.06081 | 403.979 |
| [M-H]- | Nocardamine | 2.24638071 | 8.299982885 | 0.030636987 | 599.32171 | 250.9805 |
| [M+OH]- | L-gulono-1,4-lactone | 4.27301971 | 7.979424421 | 0.017582821 | 195.05081 | 387.357 |
| [M-H]- | Daidzein 4'-sulfate | 3.353146337 | 7.682521703 | 0.026391494 | 333.00686 | 33.368 |
| [M-H-CO]- | Silybin | 3.379173734 | 7.519068685 | 0.016871067 | 453.121 | 32.793 |
| [M-H]- | Ethyl sulfate | 1.882300315 | 7.075238951 | 0.013742364 | 124.99066 | 38.071 |
| [M-H]- | D-glucono-1,5-lactone | 2.162528064 | 6.961199617 | 0.041882422 | 177.04022 | 269.735 |
| [M-H]- | Glutamic acid | 2.757577744 | 6.456080113 | 0.022887691 | 146.04552 | 305.541 |
| [M-H]- | N-Acetyl-D-Glucosamine 6-Phosphate | 1.960618925 | 5.565645449 | 0.016075125 | 300.03973 | 165.836 |
| [M-H]- | Quinate | 3.173086907 | 5.276268253 | 0.002457568 | 191.05593 | 345.186 |
| [M-H]- | D-gluconate | 2.659116859 | 5.19174649 | 0.019365628 | 195.05076 | 403.1605 |
| [M-H]- | D-glucose 6-phosphate | 1.414327393 | 4.969269841 | 0.002281942 | 259.01303 | 221.266 |
| [M-H]- | Mestranol | 3.184536415 | 4.520785838 | 0.023116179 | 309.17386 | 28.567 |
| [M-H]- | Thymol-beta-d-glucoside | 7.460247462 | 4.279160297 | 0.026941403 | 311.16875 | 31.1765 |
| [M-H]- | Hydroquinidine | 7.062729125 | 4.241633805 | 0.041148851 | 325.1841 | 30.8455 |
| [M-H]- | D-fructose | 4.997388923 | 4.148947957 | 0.019689402 | 179.0559 | 267.029 |
| [M-H]- | Malate | 4.480703243 | 4.081670984 | 0.000714104 | 133.0138 | 412.073 |
| [M-H]- | Allantoin | 1.09491069 | 3.815889326 | 0.006103372 | 157.03613 | 191.838 |
| [M-H]- | D-psicose | 1.780577195 | 3.667293195 | 0.002519831 | 179.05579 | 352.57 |
| [M-H-H2O]- | D-glucarate | 2.369376541 | 3.646624883 | 0.008140489 | 191.01949 | 263.965 |
| [M-H]- | 1,2-propanediol, 3-(1,3-benzodioxol-5-yl)- | 1.835589385 | 3.597884209 | 0.030419397 | 195.05076 | 424.711 |
| [M-H]- | 3-Methoxy-4-Hydroxyphenylglycol Sulfate | 2.906884199 | 3.453605621 | 0.003080449 | 263.02291 | 50.099 |
| [M+Cl]- | Daidzin | 1.369437374 | 3.19764134 | 0.020138972 | 451.07022 | 29.9705 |
| [M-H]- | Ostruthin | 3.338982768 | 3.078226319 | 0.017149102 | 297.15289 | 31.573 |
| [2M-H]- | 1-hexadecyl lysophosphatidic acid | 1.616796466 | 3.018187457 | 0.01807796 | 791.53366 | 114.485 |
| [M-H]- | Fructose 1-phosphate | 1.421672292 | 3.015297341 | 0.003847204 | 259.01333 | 294.11 |
| [M-H]- | D-fructose 6-phosphate | 2.258318756 | 2.813612219 | 0.021406839 | 259.01326 | 160.869 |
| (M-H)- | 3-Hydorxy-3-methylglutaric acid | 1.562509384 | 2.666943299 | 0.004385152 | 161.04528 | 267.0645 |
| [M+Cl]- | Propanoic acid, 3-[[[2-[(aminoiminomethyl)amino]-4-thiazolyl]methyl]thio]- | 1.078059665 | 2.522737882 | 0.009073585 | 294.9937 | 476.57 |
| [M-H]- | Citrate | 2.715706195 | 2.373643405 | 0.039789284 | 191.01962 | 476.82 |
| [M-H]- | .alpha.-keto-.gamma.-(methylthio)butyric acid | 1.230742543 | 2.306569873 | 0.028454592 | 147.02939 | 77.2755 |
| [M-H]- | Demethylbellidifolin | 2.094557801 | 2.30414082 | 0.037722102 | 259.02813 | 93.0635 |
| [M-H]- | 1-palmitoyl-2-linoleoyl-sn-glycero-3-phospho-(1'-rac-glycerol) | 1.160605678 | 2.215872527 | 0.023289687 | 745.50475 | 53.547 |
| [M-H]- | 6,8-dihydroxy-2,2,4,4-tetramethyl-7-(3-methylbutanoyl)-9-(2-methylpropyl)-9h-xanthene-1,3-dione | 1.03319483 | 2.032471946 | 0.023158063 | 441.2529 | 31.085 |
| [M-H]- | 2-keto-3-deoxyoctonic acid | 1.105916873 | 2.031448295 | 0.005609506 | 237.06122 | 372.1835 |
| [M-H]- | 6,8-diprenylnaringenin | 1.242469646 | 1.740185491 | 0.037999777 | 407.20909 | 99.305 |
| [M-H]- | Propionic acid | 2.406980771 | 0.636949416 | 0.044235688 | 73.02932 | 172.82 |
| [M-H]- | Corticosterone | 1.069205107 | 0.528333318 | 0.014060283 | 345.20983 | 92.0755 |
| [M-H]- | 5.beta.-androstan-3.alpha.-ol-17-one sulfate | 5.632922694 | 0.399757525 | 0.038920948 | 369.17338 | 33.0335 |
| [M-H]- | Biocytin | 2.481678326 | 0.343132465 | 0.027963888 | 371.18864 | 35.274 |
| PB vs. HP | | | | | | |
| **adduct** | **Name** | **VIP** | **Fold change** | **p-value** | **m/z** | **rt(s)** |
| [M-H]- | Taurine | 3.348802134 | 2.981910563 | 0.000501443 | 124.00686 | 300.6355 |
| [M-H]- | Zinniol | 8.788444198 | 4.44361654 | 0.000888085 | 265.14784 | 28.5455 |
| [M+Cl]- | 1-lignoceroyl-2-hydroxy-sn-glycero-3-phosphocholine | 2.224981853 | 2.336545223 | 0.001996841 | 642.40477 | 158.882 |
| [M-H]- | Cinchonine | 6.414515761 | 3.641896357 | 0.002078787 | 293.17902 | 28.128 |
| (M-H)- | 3-Hydorxy-3-methylglutaric acid | 1.500399065 | 2.501876673 | 0.002992527 | 161.04528 | 267.0645 |
| [M-H]1- | Prostaglandin f2.alpha. | 1.173403597 | 0.338275692 | 0.003085526 | 357.27923 | 41.175 |
| [M+HCO2]- | Alisol a 24-acetate | 3.415298881 | 4.076388313 | 0.004474515 | 577.37482 | 152.493 |
| [M-H]- | Methyl 3,4,5-trimethoxycinnamate | 1.637938627 | 3.600469037 | 0.006452677 | 251.10332 | 286.162 |
| [M-H-H2O]- | Leukotriene e4 | 2.121625191 | 5.351891371 | 0.007084449 | 420.24278 | 72.497 |
| [M-H]- | Gly-His-Lys | 13.04849648 | 6.285304565 | 0.007525933 | 339.1998 | 30.417 |
| [M-H-C6H12O6]- | D-turanose | 1.296638011 | 1.718510807 | 0.007656292 | 161.04529 | 307.341 |
| [M-H]- | .beta.-hydroxypropionic acid | 1.505159209 | 2.100949997 | 0.007993201 | 89.02405 | 307.893 |
| [M-H]- | Aleuritic acid | 6.426883898 | 19.41860435 | 0.00830398 | 303.19961 | 33.927 |
| [M-H]- | Madecassic acid | 1.045439511 | 0.439861666 | 0.008481277 | 503.33717 | 158.722 |
| [M-H]- | Malate | 3.079064602 | 3.779295347 | 0.008605351 | 133.0138 | 412.073 |
| [M-H]- | 15-cyclohexylpentanorprostaglandin f2.alpha. | 1.688061817 | 3.211793914 | 0.00885049 | 365.23572 | 29.609 |
| [M-H]- | Hydroquinidine | 15.02556199 | 6.478247017 | 0.009297374 | 325.1841 | 30.8455 |
| [M-H]- | 9,10-dihydroxy-12z-octadecenoic acid | 1.973706355 | 1.507078381 | 0.010194528 | 313.23839 | 95.097 |
| [M-H]- | 5-isoxazoleacetic acid, 4,5-dihydro-3-(4-hydroxyphenyl)-, methyl ester | 2.36350493 | 2.368410093 | 0.010465772 | 234.07948 | 82.653 |
| [M-H]- | 3-hydroxystanozolol glucuronide | 1.264134297 | 0.507151858 | 0.011551536 | 519.27865 | 29.0685 |
| [M-H]- | 5-(1,2,4a,5-tetramethyl-7-oxo-3,4,8,8a-tetrahydro-2h-naphthalen-1-yl)-3-methylpentanoic acid | 5.758575893 | 9.603583299 | 0.011600062 | 319.22912 | 36.225 |
| [M-H]- | Thymol-beta-d-glucoside | 14.39651956 | 5.956352075 | 0.012255465 | 311.16875 | 31.1765 |
| [M-H]- | 11-dehydrothromboxane b2 | 1.873706411 | 0.240581269 | 0.012342663 | 367.21236 | 90.852 |
| [M-H]- | Mestranol | 5.882840264 | 6.040380484 | 0.016211609 | 309.17386 | 28.567 |
| [M-H]- | Ostruthin | 5.145812001 | 3.244509778 | 0.017895482 | 297.15289 | 31.573 |
| [2M-H]- | 3-furancarboxylic acid, tetrahydro-4-methylene-2-octyl-5-oxo-, (2r,3s)- | 3.738593252 | 0.318444897 | 0.01831295 | 507.31422 | 30.524 |
| [M-H]- | 15(r),19(r)-hydroxyprostaglandin f1.alpha. | 1.725154276 | 0.300682058 | 0.018592462 | 371.2587 | 66.2855 |
| [M-H]- | D-allose | 4.409139964 | 2.063183323 | 0.019408034 | 179.05598 | 307.82 |
| [M+Cl]- | Asiatic acid | 4.339834191 | 0.507272748 | 0.020148793 | 523.31057 | 32.5495 |
| [M-H]- | Methoxyfenozide | 1.518231805 | 0.234833466 | 0.021681007 | 367.17793 | 89.732 |
| (M-H)- | 2-Methylbenzoic acid | 1.139244588 | 0.575184959 | 0.022423654 | 135.04459 | 109.5665 |
| [M-H]- | Octadecanoic acid | 1.482961067 | 0.352219168 | 0.024749031 | 283.26407 | 110.373 |
| [M-H]- | Hymeglusin | 1.358448206 | 2.553670064 | 0.025351991 | 323.16823 | 31.8895 |
| [M-H]- | N-Acetyl-D-Glucosamine 6-Phosphate | 2.790951783 | 9.236855688 | 0.02655908 | 300.03973 | 165.836 |
| [M-H]- | Methylmalonic acid | 4.019119386 | 5.201090122 | 0.027060985 | 117.01886 | 393.401 |
| [M-H]- | 16-hydroxyhexadecanoic acid | 4.488386634 | 0.467083247 | 0.030960015 | 271.22798 | 57.1785 |
| [M-H-H2O]- | 16-phenyltetranorprostaglandin f2.alpha. | 1.735136581 | 2.07753469 | 0.032060602 | 355.19463 | 33.032 |
| (M-H2O-H)- | L-homocysteic acid | 1.377587068 | 2.402714863 | 0.033872995 | 164.00191 | 69.453 |
| (M-H)- | 3,4-Dihydroxyhydrocinnamic acid | 1.380929713 | 4.806645765 | 0.036933392 | 181.05035 | 221.108 |
| [M-H]- | 3,3-dimethylglutaric acid | 1.81677038 | 0.264261628 | 0.038650556 | 159.06605 | 256.871 |
| [M-H]- | Oleanolic acid | 2.941971206 | 0.314450367 | 0.039450518 | 455.35193 | 40.035 |
| [M-H]- | 6,8-dihydroxy-2,2,4,4-tetramethyl-7-(3-methylbutanoyl)-9-(2-methylpropyl)-9h-xanthene-1,3-dione | 1.784007451 | 2.13441595 | 0.041035803 | 441.2529 | 31.085 |
| [M-H]- | L-alaninamide, n-[2-[2-(hydroxyamino)-2-oxoethyl]-4-methyl-1-oxopentyl]-3-(2-naphthalenyl)-l-alanyl- | 18.96206323 | 2.687768445 | 0.041287417 | 455.24663 | 75.539 |
| [M-H]- | Morphine n-oxide | 1.032959181 | 1.588859763 | 0.041461058 | 300.14041 | 31.8935 |
| [M-H]- | Daidzein 4'-sulfate | 2.322205087 | 5.44286979 | 0.042404331 | 333.00686 | 33.368 |
| [M-H]- | 8-gingerol | 3.611728825 | 3.394905172 | 0.042444525 | 321.20993 | 27.9365 |
| [M-H]- | Oleanonic acid | 7.529592566 | 0.082533027 | 0.044334983 | 453.33724 | 37.856 |
| [M-H]- | Rauwolscine | 4.237013608 | 2.801111944 | 0.045512211 | 353.19943 | 29.037 |
| [M-H]- | 1h-indole-3-propanoic acid | 1.853388918 | 0.571775143 | 0.046387453 | 188.07144 | 108.275 |
| [M-H]- | Trans-ferulic acid | 1.51308483 | 2.542149515 | 0.047069454 | 193.05036 | 165.691 |
| [M-H-C5H8O2]- | 1h-indole-1-pentanoic acid, 3-[[[1-(aminocarbonyl)-2,2-dimethylpropyl]amino]carbonyl]- | 1.214473035 | 8.244063556 | 0.049351421 | 272.12926 | 42.954 |
| PB vs. P | | | | | | |
| **adduct** | **Name** | **VIP** | **Fold change** | **p-value** | **m/z** | **rt(s)** |
| [M-H]- | Benzoic acid | 1.680193221 | 0.443707382 | 0.001884892 | 121.02879 | 127.4975 |
| [M-H]- | Biocytin | 3.599344931 | 3.172244195 | 0.001949106 | 371.18864 | 35.274 |
| [M-H]- | 5.beta.-androstan-3.alpha.-ol-17-one sulfate | 6.557939453 | 2.900713193 | 0.004282649 | 369.17338 | 33.0335 |
| [M-H]- | Quinate | 1.455844085 | 0.362811164 | 0.039252262 | 191.05593 | 345.186 |
| [M-H]- | 2-Hydroxyphenylacetic acid | 1.017772558 | 0.50142265 | 0.006380427 | 151.04076 | 53.324 |
| [M-H]- | 3,3-dimethylglutaric acid | 1.681157746 | 0.332801967 | 0.013774625 | 159.06605 | 256.871 |
| [M-H]- | 11-dehydrothromboxane b2 | 1.087285288 | 0.372599214 | 0.021823591 | 367.21236 | 90.852 |
| [M-H]- | Eplerenone hydroxy acid | 1.804517879 | 2.190844999 | 0.022393162 | 431.21081 | 71.51 |
| [M-H]- | 3-Methoxy-4-Hydroxyphenylglycol Sulfate | 3.174853095 | 0.259107401 | 0.003582272 | 263.02291 | 50.099 |
| [M-H]- | Mesoporphyrin ix | 2.681327564 | 0.268117586 | 0.024981904 | 565.28462 | 137.8345 |
| [M-H]- | 1,2-propanediol, 3-(1,3-benzodioxol-5-yl)- | 1.721596256 | 0.205156794 | 0.024078796 | 195.05076 | 424.711 |
| [M-H-H2O]- | Leukotriene e4 | 1.948995299 | 3.602900895 | 0.026805387 | 420.24278 | 72.497 |
| [M-H]- | D-gluconate | 2.386902382 | 0.172475162 | 0.027773458 | 195.05076 | 403.1605 |
| [M-H]- | 3-hydroxyoctanoic acid | 1.016437226 | 0.266562798 | 0.029055152 | 159.09186 | 263.2075 |
| [M-H]- | 3-dehydroepiandrosterone sulfate | 10.22131435 | 2.387784012 | 0.029286734 | 367.15811 | 31.463 |
| [M-H]- | Ethyl sulfate | 1.292556755 | 0.136934342 | 0.025845551 | 124.99066 | 38.071 |
| [M-H]- | 3-hydroxy-3-methylglutaric acid | 1.039406232 | 0.503985427 | 0.03471771 | 161.04526 | 379.227 |
| [M+OH]- | L-gulono-1,4-lactone | 3.906341727 | 0.113596458 | 0.029565625 | 195.05081 | 387.357 |
| [M-H]- | (S)-2-Hydroxyglutarate | 1.076243919 | 0.657385155 | 0.04045228 | 147.0298 | 399.284 |
| [M-H-CO2]- | Indole-3-butyric acid | 7.00386781 | 0.101097236 | 0.041676265 | 158.08199 | 316.038 |
| [M-H]- | Hydroquinone | 1.182898599 | 0.18435114 | 0.045704589 | 109.02903 | 27.195 |
| [M-H]- | 1-palmitoyl-2-hydroxy-sn-glycero-3-phospho-(1'-rac-glycerol) | 4.160057931 | 0.455856735 | 0.047938083 | 483.27268 | 164.5735 |
| [M-H]- | 7-hydroxyquetiapine | 1.003745988 | 1.800306683 | 0.049819822 | 398.16887 | 59.0815 |

| Table S2. Fecal metabolites with significant differences under ESI+ mode between groups | | | | | | |
| --- | --- | --- | --- | --- | --- | --- |
| P vs. HP | | | | | | |
| **adduct** | **Name** | **VIP** | **Fold change** | **p-value** | **m/z** | **rt(s)** |
| [M+H]+ | Phenylacetyl-l-glutamine | 1.766484913 | 8.213373225 | 0.015749177 | 265.11733 | 221.642 |
| [M+H]+ | Methyl (1-(cyclohexylmethyl)-1h-indole-3-carbonyl)-l-valinate | 3.910324635 | 7.828083734 | 0.000963856 | 371.22721 | 90.3935 |
| [M+Na]+ | 2-[[7-hydroxy-1-(4-hydroxy-3,5-dimethoxyphenyl)-3-(hydroxymethyl)-6,8-dimethoxy-1,2,3,4-tetrahydronaphthalen-2-yl]methoxy]-6-(hydroxymethyl)oxane-3,4,5-triol | 1.219482562 | 5.455417403 | 0.043481246 | 605.2173 | 162.7235 |
| [M+H]+ | Myristoyl-l-carnitine | 1.337484823 | 3.489886992 | 0.019805157 | 372.30849 | 164.909 |
| [M+H]+ | Arcaine | 1.274706385 | 3.468690558 | 0.048628721 | 173.13827 | 282.353 |
| [M+H]+ | 1-stearoyl-2-hydroxy-sn-glycero-3-phosphocholine | 4.918450091 | 3.449939686 | 0.041422255 | 524.36892 | 178.774 |
| [M+H-NH3]+ | N-(1-amino-3,3-dimethyl-1-oxobutan-2-yl)-1-pentyl-1h-indole-3-carboxamide | 11.36209907 | 3.247388531 | 0.003259827 | 327.20078 | 81.893 |
| [M+H]+ | Dibucaine | 12.02224443 | 3.164076509 | 0.003806625 | 344.22717 | 81.643 |
| [M+H-2H2O]+ | N-acetylsphingosine | 3.160299437 | 3.114479715 | 0.015213858 | 306.27766 | 34.908 |
| [M+H]+ | 2-dimethylamino-6-hydroxypurine | 1.058272401 | 2.954005825 | 0.009759409 | 180.08658 | 135.0895 |
| [M+H]+ | 2-(2-butoxyethoxy)acetic acid | 2.965319335 | 2.868958597 | 0.015515584 | 177.11075 | 68.334 |
| [M+H]+ | Betaine | 17.57192332 | 2.739920259 | 0.001233787 | 118.086 | 264.934 |
| [M+H]+ | Hc toxin | 2.256666816 | 2.727503771 | 0.005257108 | 437.23259 | 96.818 |
| [M+H-H2O]+ | 17-trifluoromethylphenyltrinorprostaglandin f2.alpha. methyl ester | 1.204723381 | 2.71456847 | 0.003816236 | 453.2065 | 97.6545 |
| [M+H]+ | Linoleoyl ethanolamide | 6.549332568 | 2.691808743 | 0.035282625 | 324.28844 | 34.457 |
| (M+H)+ | Ethyl 3-hydroxybutyrate | 3.500445534 | 2.624136139 | 0.001585444 | 133.08473 | 63.214 |
| [M+H]+ | Tetraethylene glycol | 4.332484423 | 2.557777577 | 0.001716646 | 195.12221 | 61.01 |
| [M+H]+ | .alpha.-linolenoyl ethanolamide | 1.416705198 | 2.460312106 | 0.011228602 | 322.2721 | 34.175 |
| [M+H-H2O]+ | 17-trifluoromethylphenyltrinorprostaglandin f2.alpha. isopropyl ester | 2.06775525 | 2.444207775 | 0.005356785 | 481.25881 | 105.1195 |
| [M+Na]+ | Octaethylene glycol | 2.099403923 | 2.419334993 | 0.00595263 | 393.20664 | 89.1375 |
| [M+H]+ | 2-piperidinecarboxylic acid, 1-(3,3-dimethyl-1,2-dioxopentyl)-, (1r)-1-(3-aminophenyl)-3-(3,4-dimethoxyphenyl)propyl ester, (2s)- | 1.917119626 | 2.247844965 | 0.013495407 | 525.28496 | 110.9655 |
| [M+H]+ | Trigonelline | 2.373539161 | 2.186249216 | 0.019879179 | 138.05419 | 283.845 |
| [M+H]+ | Isobutyric acid | 1.445534426 | 2.174545546 | 0.003420685 | 89.05888 | 61.8 |
| [M+H]+ | Mifepristone | 1.235552656 | 2.17250453 | 0.005862376 | 430.26577 | 170.21 |
| [M+Na]+ | 2,3-dinor-8-isoprostaglandin-f2.alpha. | 2.000786908 | 2.099289951 | 0.010786841 | 349.18089 | 82.601 |
| [M]+ | 7,8-didehydroastaxanthin | 1.009219351 | 1.969197743 | 0.04690168 | 594.36567 | 86.489 |
| [M+H]+ | 2-aminobenzamide | 1.107826182 | 1.952438747 | 0.038830041 | 137.06931 | 46.614 |
| [M+H-C3H7O5P]+ | Glycerophosphocholine | 6.69312004 | 1.703407985 | 0.031630363 | 104.10709 | 260.395 |
| [M+H]+ | Fenpropidin | 4.551799883 | 0.692642154 | 0.007369509 | 274.27371 | 60.342 |
| [M+H]+ | C17-sphinganine | 1.73428657 | 0.690569239 | 0.000227697 | 288.28809 | 36.825 |
| [M+H]+ | Nicotinate | 2.005285295 | 0.620728641 | 0.031632711 | 124.03849 | 209.797 |
| [M+H]+ | Hypoxanthine | 6.302822016 | 0.552332264 | 0.02476226 | 137.04586 | 160.5855 |
| [M+H-H2O]+ | Carbofuran phenol-3-ketone | 1.018095404 | 0.55226381 | 0.020863752 | 161.05856 | 37.167 |
| (M+NH4)+ | Benzamide | 1.406245674 | 0.5248214 | 0.044665258 | 139.08549 | 47.937 |
| [M+H]+ | Guaiacol methyl ether | 1.496351009 | 0.508891413 | 0.023103262 | 139.08531 | 137.835 |
| [M+H-2H2O]+ | 4.alpha.-hydroxystanozolol | 4.740222317 | 0.490883797 | 0.038319026 | 309.2567 | 116.5775 |
| [M+H]+ | Cys-Trp-Arg | 1.061617667 | 0.42943388 | 0.031103545 | 464.21503 | 229.2795 |
| [M+Na]+ | Palmitoyl 3-carbacyclic phosphatidic acid | 3.758160942 | 0.418147894 | 0.017807482 | 413.26432 | 116.372 |
| [M+H-C7H6N2]+ | 1,5-naphthyridine, 2-[3-(6-methyl-2-pyridinyl)-1h-pyrazol-4-yl]- | 1.25189416 | 0.351884247 | 0.017957106 | 170.05558 | 39.4045 |
| [M+H-CH6O2]+ | Lappaconitine | 1.486409838 | 0.348171074 | 0.032794995 | 535.26795 | 35.764 |
| (M+H)+ | Cyclohexylamine | 4.009630411 | 0.289296434 | 0.040504798 | 100.11085 | 71.223 |
| [M+Na]+ | Tributyrin | 1.280177868 | 0.278975451 | 0.008570627 | 325.15231 | 110.496 |
| [M+H]+ | 4-cholestenone | 4.771026109 | 0.271981879 | 0.01935748 | 385.34483 | 30.934 |
| [M+H]+ | 1,2-dipentadecanoyl-sn-glycero-3-phosphoethanolamine | 1.429407448 | 0.238501128 | 0.037740668 | 664.48681 | 142.808 |
| [M+H]+ | 2-mohma [dmed-fahfa] | 1.210623582 | 0.235273701 | 0.045161 | 523.4682 | 142.228 |
| [M+H]+ | Clorgyline | 1.033048824 | 0.176248015 | 0.021916845 | 272.04708 | 141.489 |
| PB vs. HP | | | | | | |
| **adduct** | **Name** | **VIP** | **Fold change** | **p-value** | **m/z** | **rt(s)** |
| [M+H]+ | Fenpropidin | 5.989548717 | 0.4875186 | 1.52463E-05 | 274.27371 | 60.342 |
| [M+H-C6H12O]+ | Zerumbone | 1.532047647 | 0.683328542 | 0.000515206 | 119.08433 | 34.1265 |
| [M+H]+ | Lauroyl-l-carnitine | 1.80309238 | 4.061061199 | 0.003003541 | 344.27739 | 172.6475 |
| (M+NH4)+ | alpha-Linolenic acid | 1.792161851 | 0.623653975 | 0.004884096 | 296.25662 | 34.421 |
| (2M+H)+ | Lavandulol | 2.389842229 | 9.275427799 | 0.006581417 | 309.27666 | 32.1685 |
| [M+H-H2O]+ | 3-methylbenzyl alcohol | 1.173833499 | 0.730894323 | 0.006859815 | 105.06871 | 35.621 |
| [M+H]+ | Myristoyl-l-carnitine | 1.696878424 | 3.799759565 | 0.007023898 | 372.30849 | 164.909 |
| [M+H]+ | 5z,8z,14z-eicosatrienoic acid | 1.127974546 | 3.158975242 | 0.010105675 | 307.26081 | 33.1275 |
| [M+H]+ | C17-sphinganine | 1.694661812 | 0.721007164 | 0.010798678 | 288.28809 | 36.825 |
| [M+H-H2O]+ | Sumaresinolic acid | 1.283318148 | 0.307007533 | 0.011997374 | 455.34896 | 58.551 |
| [M+H]+ | Creatinine | 6.758343325 | 3.623794895 | 0.012363202 | 114.06524 | 161.703 |
| [M+H-C18H34O2]+ | N-palmitoyl-d-sphingosine | 3.085866968 | 0.807948423 | 0.012374557 | 256.26268 | 33.695 |
| [M+H]+ | N-Stearoylsphingosine (Ceramide C18) | 1.000868066 | 0.539235878 | 0.012530087 | 566.54901 | 33.175 |
| [M+H]+ | Hypoxanthine | 6.298184526 | 0.526047746 | 0.012857944 | 137.04586 | 160.5855 |
| [M+H]+ | 4-hydroxy-1-(2-hydroxyethyl)-2,2,6,6-tetramethylpiperidine | 1.098089853 | 0.474440259 | 0.013030023 | 202.17868 | 106.11 |
| (M+H)+ | NG,NG-dimethyl-L-arginine(ADMA) | 1.098869577 | 1.432948152 | 0.013880765 | 203.14938 | 575.581 |
| [M+H]+ | Methyl (1-(cyclohexylmethyl)-1h-indole-3-carbonyl)-l-valinate | 3.857519678 | 7.57532668 | 0.014168603 | 371.22721 | 90.3935 |
| [M+NH4]+ | Beta-octylglucoside | 1.489791926 | 2.939216673 | 0.014489103 | 310.19962 | 191.5275 |
| [M+Na]+ | 1alpa,3beta-diacetoxy-22-hopanol | 2.077668432 | 0.636458355 | 0.014689841 | 567.41656 | 32.2315 |
| [M+H-CH5N]+ | 4-(methylamino)-4-(3-pyridyl)butyric acid | 1.623393872 | 0.467917048 | 0.015277833 | 164.06953 | 243.8 |
| [M+H]+ | Glufosinate | 2.434820607 | 0.53191333 | 0.015634495 | 182.08018 | 242.665 |
| [M+H]+ | Avocadyne acetate | 1.02899437 | 2.020736212 | 0.016947624 | 327.25148 | 31.564 |
| [M+H]+ | Veratramine | 2.28118509 | 3.060246871 | 0.020607867 | 410.28813 | 170.814 |
| [M+H]+ | L-palmitoylcarnitine | 3.396287746 | 2.551209641 | 0.023771601 | 400.34024 | 162.105 |
| (M+CH3CN+H)+ | Isocaproic acid | 4.945666343 | 0.184958501 | 0.024718203 | 158.1167 | 38.4615 |
| [M+H-C7H6N2]+ | 1,5-naphthyridine, 2-[3-(6-methyl-2-pyridinyl)-1h-pyrazol-4-yl]- | 1.516303038 | 0.365559991 | 0.025207969 | 170.05558 | 39.4045 |
| [M+H]+ | Biliverdin | 2.03967666 | 2.139044879 | 0.027116166 | 583.25259 | 227.841 |
| [M+H]+ | Clorgyline | 1.108402301 | 0.209833178 | 0.027409391 | 272.04708 | 141.489 |
| [M+H]+ | 4-aminovaleric acid betaine | 24.28445707 | 0.474695764 | 0.0277307 | 160.13339 | 373.487 |
| [M+H-CH4O]+ | Dimethyl azelate | 1.216736551 | 0.447364398 | 0.027901982 | 185.12787 | 43.766 |
| [M+H]+ | 2-(2-butoxyethoxy)acetic acid | 2.841597937 | 2.855435814 | 0.02806353 | 177.11075 | 68.334 |
| [M+H]+ | Myriocin | 3.108186451 | 5.088981304 | 0.028232673 | 402.28263 | 207.323 |
| [M+H]+ | Oleamide | 11.98181724 | 0.806646354 | 0.028806934 | 282.28029 | 33.2255 |
| [M+H]+ | Cyclopamine | 1.938615584 | 3.123602293 | 0.03053478 | 412.30304 | 174.144 |
| [M+H]+ | 1-oleoyl-sn-glycero-3-phosphoethanolamine | 2.598157721 | 0.328816903 | 0.031180073 | 480.30606 | 185.097 |
| [M+Na]+ | Octaethylene glycol | 2.008254935 | 2.294575005 | 0.031540148 | 393.20664 | 89.1375 |
| [M+H]+ | 4-nitrosodiphenylamine | 1.729619635 | 0.448114016 | 0.032711181 | 199.10611 | 117.541 |
| [M+H]+ | (-)-riboflavin | 1.152747202 | 0.599976951 | 0.032876143 | 377.14353 | 202.553 |
| [M+H]+ | Guaiacol methyl ether | 1.110277429 | 0.551846234 | 0.035082447 | 139.08531 | 137.835 |
| [M+H]+ | 3-methylhistamine | 2.296990511 | 0.237634261 | 0.035716787 | 126.09022 | 44.377 |
| [M+H-NH3]+ | N-(1-amino-3,3-dimethyl-1-oxobutan-2-yl)-1-pentyl-1h-indole-3-carboxamide | 10.13378607 | 2.819684679 | 0.036932901 | 327.20078 | 81.893 |
| [M+H]+ | Betonicine | 1.699321051 | 0.303436877 | 0.037996117 | 160.07452 | 221.144 |
| (M+Na)+ | 25-hydroxyvitamin D3 | 1.289866606 | 0.343296782 | 0.038186926 | 423.3205 | 48.891 |
| (M+H-H2O)+ | Sphinganine | 2.797886143 | 0.799098203 | 0.040302346 | 284.29241 | 33.2255 |
| [M+H]+ | Dibucaine | 10.69047631 | 2.721857224 | 0.041450179 | 344.22717 | 81.643 |
| [M+H-H2O]+ | Trans-trans-10,11-epoxyfarnesenic acid methyl ester | 1.024236449 | 0.641048359 | 0.041907939 | 249.18332 | 34.089 |
| [M+Na]+ | Proscillaridin a | 1.677183746 | 0.277771507 | 0.041921604 | 553.27659 | 121.589 |
| [M+H-H2O]+ | .alpha.-ionone | 1.015813223 | 0.784827988 | 0.043601193 | 175.14667 | 32.125 |
| (M+H)+ | Ethyl 3-hydroxybutyrate | 2.980463991 | 2.255354923 | 0.044295233 | 133.08473 | 63.214 |
| [M+H-H2O]+ | 17-trifluoromethylphenyltrinorprostaglandin f2.alpha. methyl ester | 1.14556177 | 2.414676166 | 0.04432878 | 453.2065 | 97.6545 |
| [M+H-C8H10O3]+ | Rotenone | 1.113053508 | 0.562114147 | 0.044458603 | 241.09569 | 179.889 |
| [M+H]+ | Isobutyric acid | 1.32029173 | 1.937036765 | 0.045677618 | 89.05888 | 61.8 |
| [M+H]+ | Celaxanthin | 3.987809489 | 0.541531033 | 0.046269641 | 551.42271 | 31.722 |
| [M+H]+ | Valine betaine | 1.718267589 | 0.407092083 | 0.048412147 | 160.1319 | 218.563 |
| PB vs. P | | | | | | |
| **adduct** | **Name** | **VIP** | **Fold change** | **p-value** | **m/z** | **rt(s)** |
| (M+H)+ | L-Tyrosine | 1.124997219 | 0.434921218 | 0.001922066 | 182.08016 | 363.319 |
| (M+NH4)+ | alpha-Linolenic acid | 1.292280437 | 0.653773516 | 0.00425727 | 296.25662 | 34.421 |
| [M+H]+ | Trigonelline | 3.007366424 | 0.378027272 | 0.00426738 | 138.05419 | 283.845 |
| [M+H]+ | Naringin dihydrochalcone | 1.029694429 | 4.421250588 | 0.011318237 | 583.21753 | 359.7945 |
| [M+Na]+ | Ht-2 toxin | 1.013059116 | 3.407729316 | 0.014308385 | 447.21283 | 399.101 |
| [M+H]+ | Betaine | 15.37404912 | 0.503744169 | 0.014949984 | 118.086 | 264.934 |
| [M+H]+ | [6]-shogaol | 1.07537203 | 1.751983495 | 0.01709592 | 277.17842 | 71.8015 |
| [M+H]+ | Fenpropidin | 2.088344672 | 0.703853494 | 0.019033832 | 274.27371 | 60.342 |
| [M+H]+ | Celaxanthin | 3.568221113 | 0.543227182 | 0.020079171 | 551.42271 | 31.722 |
| [M+H-C18H34O2]+ | N-palmitoyl-d-sphingosine | 1.704034497 | 0.808412182 | 0.024149424 | 256.26268 | 33.695 |
| [M+H]+ | 7,3',4'-trihydroxyisoflavone | 1.738285743 | 4.001668333 | 0.024570656 | 271.07958 | 62.934 |
| [M+H]+ | Trp-Arg-Arg | 1.023745872 | 0.471385589 | 0.026470194 | 517.30971 | 247.375 |
| (M+H)+ | Cyclohexylamine | 5.697031322 | 3.784354688 | 0.03088384 | 100.11085 | 71.223 |
| [M+2H]2+ | 1,3-bis(tris(hydroxymethyl)methylamino)propane | 1.714497614 | 3.484353005 | 0.031699203 | 142.12137 | 257.516 |
| [M+H]+ | Pyridoxine | 1.814613949 | 0.466679021 | 0.036350333 | 170.07964 | 108.1925 |
| [M+H]+ | (-)-riboflavin | 1.146469621 | 0.548131708 | 0.037075567 | 377.14353 | 202.553 |
| [M+Na]+ | Palmitoyl 3-carbacyclic phosphatidic acid | 3.366001471 | 1.936188022 | 0.038522023 | 413.26432 | 116.372 |
| [M+H-C5H13SO3N]+ | 3-[(cholamidopropyl)dimethylammonio]-1-propanesulfonate | 1.436973824 | 0.214076034 | 0.038659532 | 448.34011 | 153.897 |
| [M+H-CH5N]+ | N-methyltryptamine | 1.991903221 | 3.595448608 | 0.039314451 | 144.07967 | 182.668 |
| [M+H]+ | Urobilin | 13.69485844 | 2.511595335 | 0.039766311 | 591.31714 | 273.903 |
| [M+H]+ | Cycloate | 1.887525268 | 0.094790075 | 0.041419166 | 216.15814 | 38.497 |
| [M+H]+ | Pyroglu-Gly-Arg | 1.640855439 | 0.242116173 | 0.041871467 | 343.16086 | 212.914 |
| [M+H]+ | Caldine | 3.834293538 | 11.98209345 | 0.042898426 | 132.13676 | 261.7525 |
| [M+H]+ | 1-oleoyl-sn-glycero-3-phosphoethanolamine | 2.065674899 | 0.353023607 | 0.043529953 | 480.30606 | 185.097 |
| [M+H]+ | Arginine | 1.679088281 | 1.831559181 | 0.044428249 | 175.11877 | 526.159 |
| [M+H]+ | N-acetylcadaverine | 3.668916828 | 2.941359449 | 0.044780503 | 145.1326 | 289.928 |
| [M+H]+ | G-guanidinobutyrate | 1.588921874 | 0.2621594 | 0.045549089 | 146.09139 | 347.7515 |
| [M+H]+ | DL-Glutamic acid | 1.673351978 | 0.610186406 | 0.04650287 | 148.05956 | 383.344 |
| [M+H-NH3]+ | .alpha.-methyltryptamine | 1.093180038 | 0.486259274 | 0.048388914 | 158.09502 | 216.8665 |
| (M+H-H2O)+ | 1-Aminocyclopropanecarboxylic acid | 1.052957664 | 0.641154721 | 0.048931559 | 84.04368 | 383.236 |
