# Supplementary material for: Psychobiotic Lactobacillus plantarum JYLP-326 relieves anxiety, depression, and insomnia symptoms in test anxious college via modulating the gut microbiota and its metabolism
Source: Front Immunol. 2023 Mar 23;14:1158137. doi: 10.3389/fimmu.2023.1158137 (PMC10077425; doi:10.3389/fimmu.2023.1158137)
Supplement: Supplementary file 2 [file DataSheet_1.docx]

**Supplementary information**

Psychobiotic *Lactobacillus plantarum* JYLP-326 relieves anxiety, depression, and insomnia symptoms in test anxious college students *via* modulating the gut microbiota and its metabolism

Ruizhe Zhu ^1^, Yilin Fang ^1^, Hongyu Li ^1^, Ying Liu ^2^, Jing Wei ^1^, Shuwei Zhang ^1^, Liwei Wang ^1^, Rui Fan ^1^, Lingfang Wang ^1^, Shengjie Li ^1,^ * and Tingtao Chen ^1,^ *

^1^ National Engineering Research Center for Bioengineering Drugs and the Technologies, Institute of Translational Medicine, Nanchang University, Nanchang, China.

^2^ Institute of Life Science, Nanchang University, Nanchang, China

***** Correspondences: chentingtao1984@163.com (T-T. C.) and lishengjie1104@ncu.edu.cn (S-J. L.); Tel.: +86-791-83827165

Supplementary Figure 1


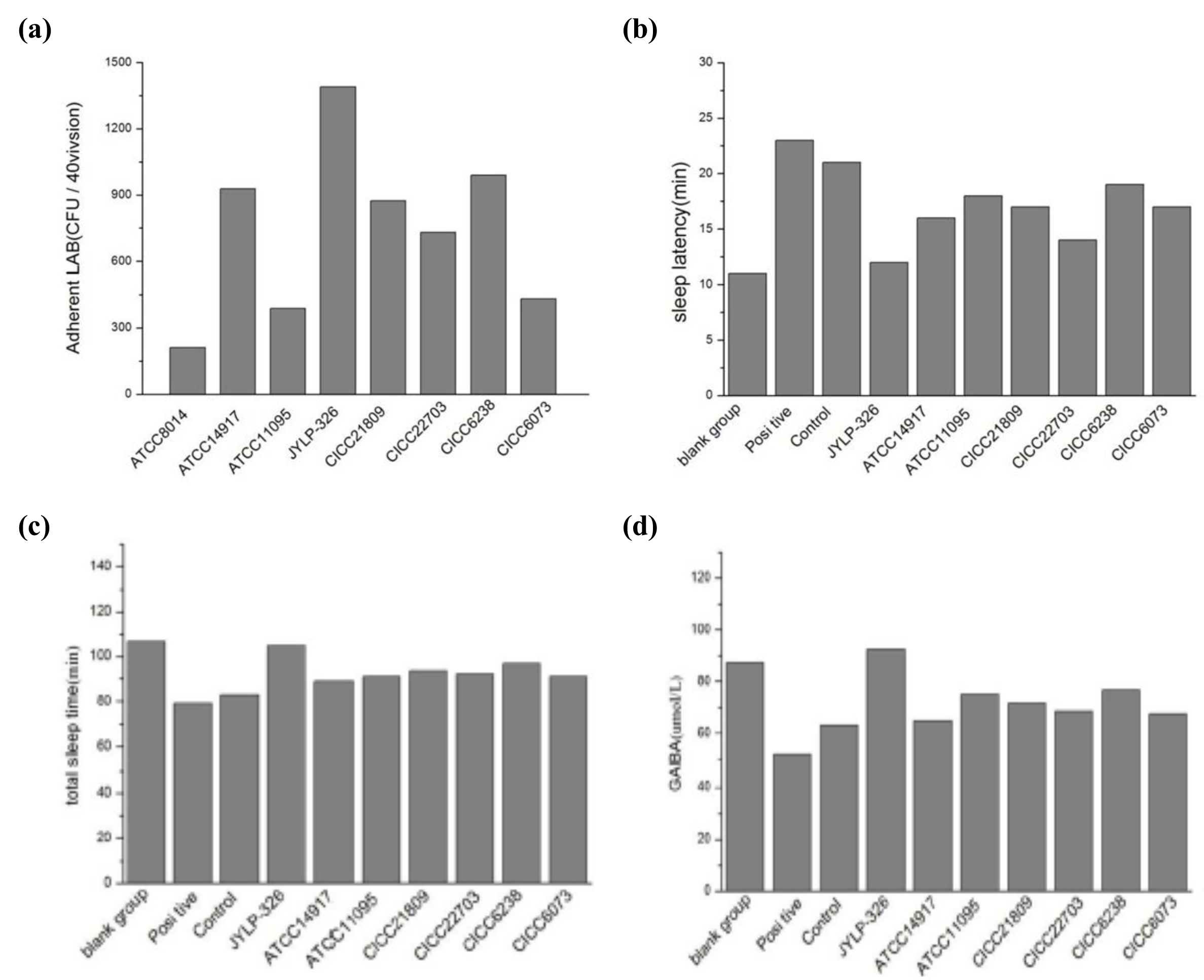


**Figure S1. Characteristics of *Lactobacillus plantarum* JYLP-326 *in vitro* and *in vivo*.** (a) The CFU of adherent bacteria to Caco-2 cell line per 40 visions. ATCC8014, ATCC14917, ACCC11095, CICC21809, CICC22703, CICC6238, and CICC6073 refer to different *L. plantarum* strains. (b) The sleep latency time and (c) total sleep time in 4-Chloro-DL-phenylalanine-induced insomnia rat model after taking probiotic strains. (d) The content of γ-aminobutyric acid in the rats’ hypothalamus after taking probiotic strains. Blank group, the normal rats treated with sterile saline; Positive group, the insomnia rats treated with sterile saline; Control, the insomnia rats treated with maltodextrin; JYLP-326, ATCC14917, ACCC11095, CICC21809, CICC22703, CICC6238, and CICC6073, the insomnia rats treated with the relevant strains at the dose of 10^10^ CFU. Data were presented as mean of three replicates. All the figures were extracted from the Chinese patent CN 110791451 A.

Supplementary Figure 2


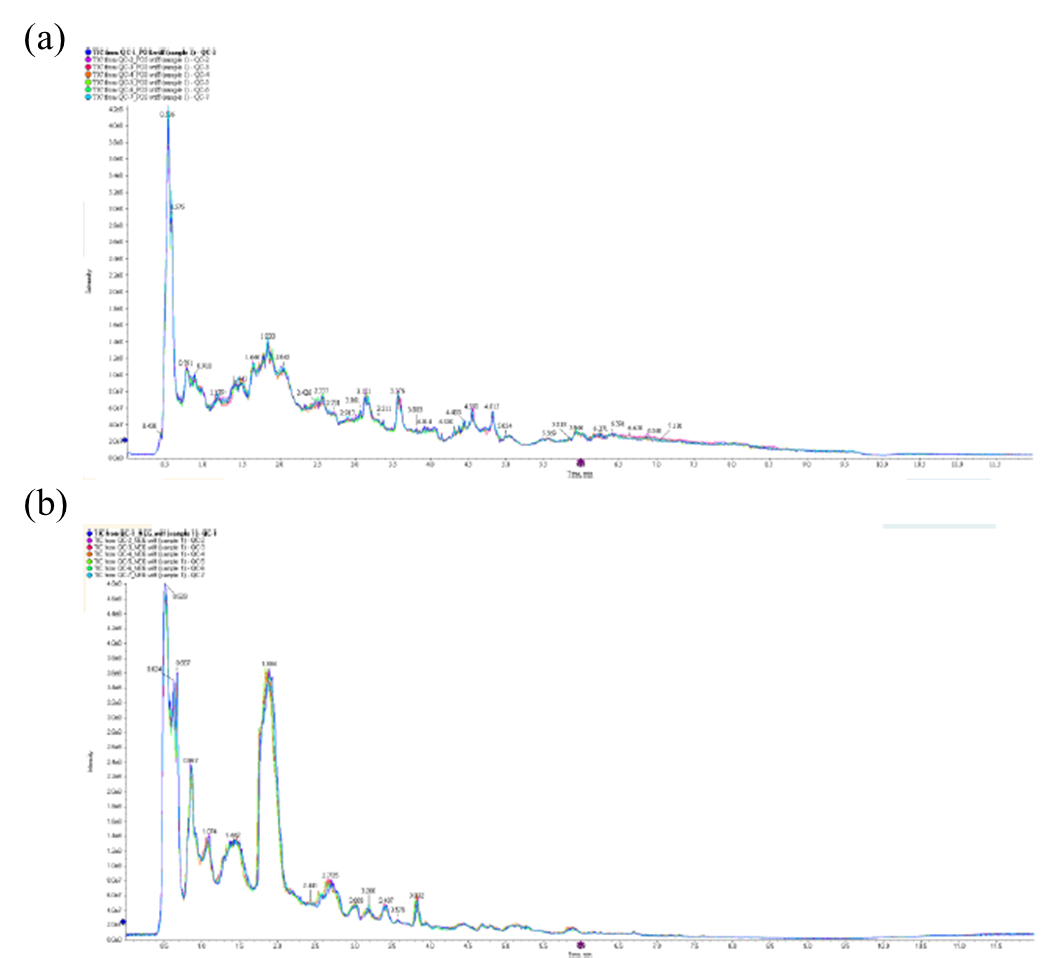


**Figure S2. The total ion chromatogram (TIC) of seven quality control (QC) samples.** (a) TIC spectra under ESI+ mode. (b) TIC spectra under ESI- mode. The response intensity and retention time of each chromatographic peaks among QC samples overlapped substantially, indicating that the variation caused by the instrument error is small during the whole experiment.

Supplementary Figure 3


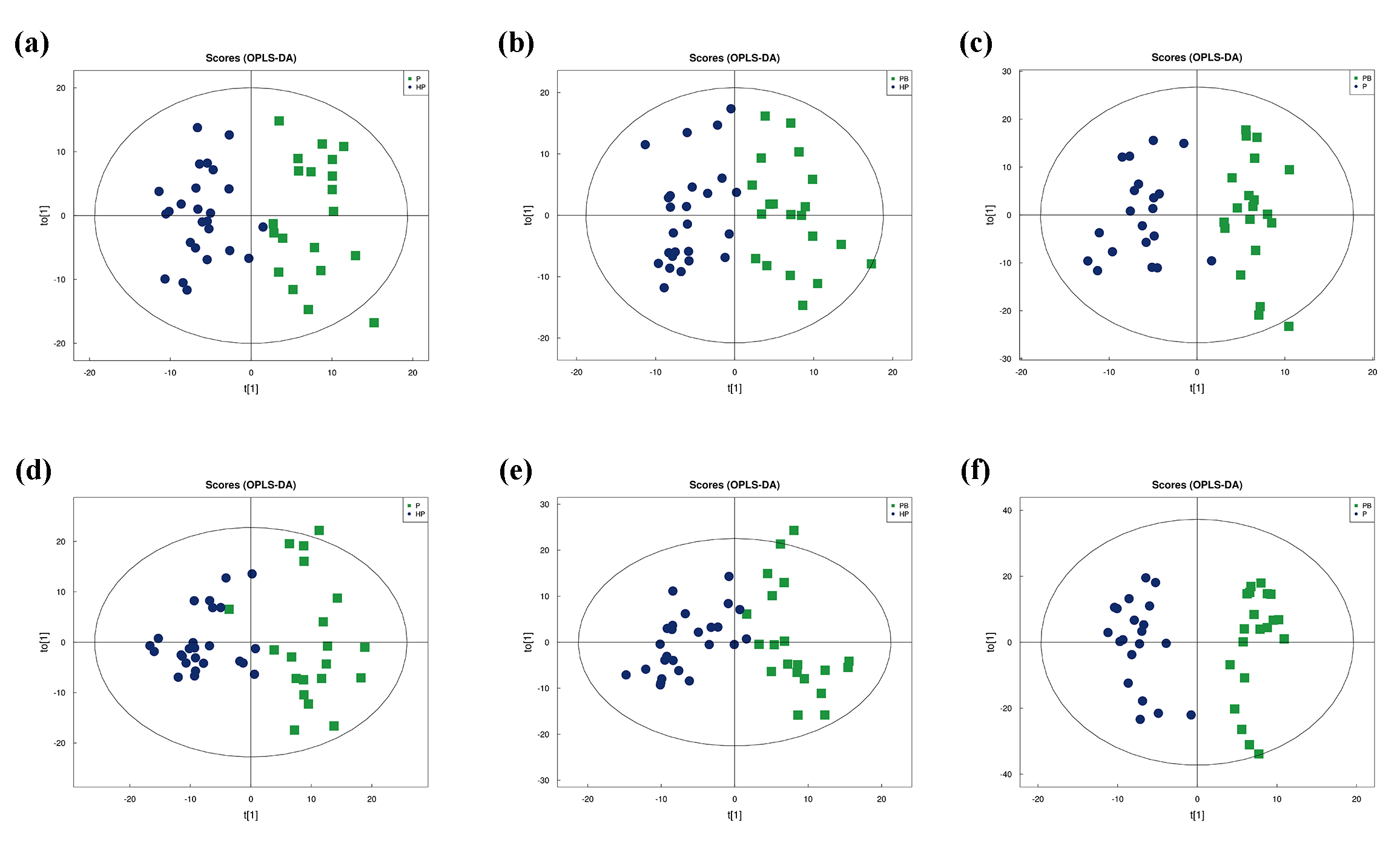


**Figure S3. OPLS-DA analysis of fecal differential metabolites among each group.** (a-c) OPLA-DA score plots under ESI- mode and (d-f) under ESI+ mode. (a and d, b and e) OPLA-DA plots in the placebo group and probiotic group in comparison to the control group; (c and f) OPLA-DA plot in the probiotic group in comparison to the placebo group.

Supplementary Figure 4


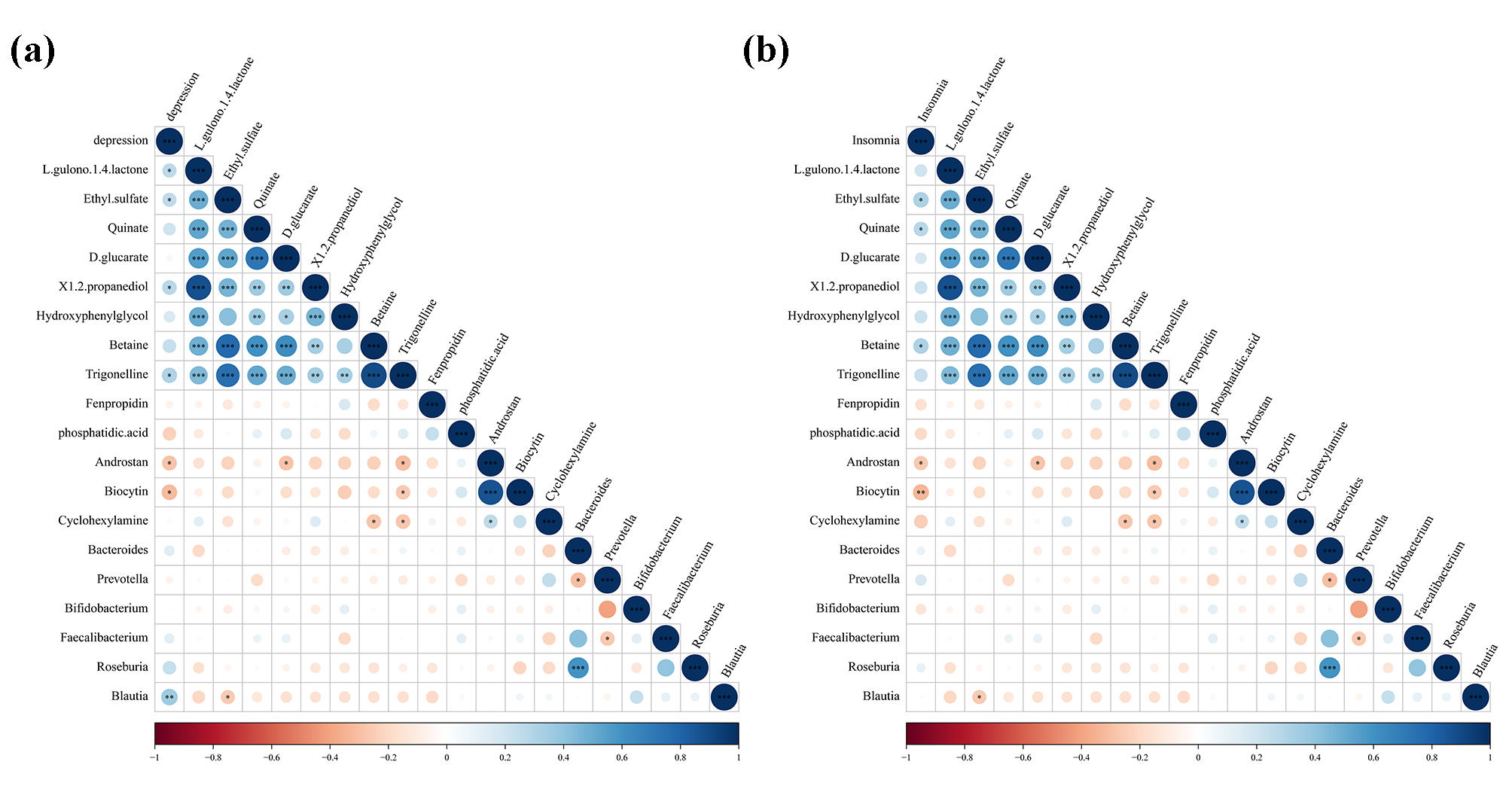


**Figure S4. Spearman correlation analysis among depression/insomnia, gut microbiota, and fecal metabolites.** Spearman’s rank correlation coefficient among depression symptom scores (a) / insomnia scores (b), 13 metabolites in table 2 and top 6 relative abundance of gut microbiota. P values are depicted in red and blue, where red refers to a negative correlation and blue refers to a positive correlation. * *p* < 0.05, ** *p* < 0.01, *** *p* < 0.001.
